# Characterization of Arabidopsis FPS Isozymes and *FPS* Gene Expression Analysis Provide Insight into the Biosynthesis of Isoprenoid Precursors in Seeds

**DOI:** 10.1371/journal.pone.0049109

**Published:** 2012-11-07

**Authors:** Verónica Keim, David Manzano, Francisco J. Fernández, Marta Closa, Paola Andrade, Daniel Caudepón, Cristina Bortolotti, M. Cristina Vega, Montserrat Arró, Albert Ferrer

**Affiliations:** 1 Department of Molecular Genetics, Centre for Research in Agricultural Genomics (CRAG) (CSIC-IRTA-UAB-UB), Campus UAB, Bellaterra (Cerdanyola del Vallès), Barcelona, Spain; 2 Department of Biochemistry and Molecular Biology, Faculty of Pharmacy, University of Barcelona, Barcelona, Spain; 3 Department of Structural and Quantitative Biology, Centre for Biological Research (CIB-CSIC), Madrid, Spain; Lawrence Berkeley National Laboratory, United States of America

## Abstract

*Arabidopsis thaliana* contains two genes encoding farnesyl diphosphate (FPP) synthase (FPS), the prenyl diphoshate synthase that catalyzes the synthesis of FPP from isopentenyl diphosphate (IPP) and dimethylallyl diphosphate (DMAPP). In this study, we provide evidence that the two Arabidopsis short FPS isozymes FPS1S and FPS2 localize to the cytosol. Both enzymes were expressed in *E. coli*, purified and biochemically characterized. Despite FPS1S and FPS2 share more than 90% amino acid sequence identity, FPS2 was found to be more efficient as a catalyst, more sensitive to the inhibitory effect of NaCl, and more resistant to thermal inactivation than FPS1S. Homology modelling for FPS1S and FPS2 and analysis of the amino acid differences between the two enzymes revealed an increase in surface polarity and a greater capacity to form surface salt bridges of FPS2 compared to FPS1S. These factors most likely account for the enhanced thermostability of FPS2. Expression analysis of *FPS::GUS* genes in seeds showed that *FPS1* and *FPS2* display complementary patterns of expression particularly at late stages of seed development, which suggests that Arabidopsis seeds have two spatially segregated sources of FPP. Functional complementation studies of the Arabidopsis *fps2* knockout mutant seed phenotypes demonstrated that under normal conditions FPS1S and FPS2 are functionally interchangeable. A putative role for FPS2 in maintaining seed germination capacity under adverse environmental conditions is discussed.

## Introduction

All isoprenoids are derived from the C_5_ building blocks isopentenyl diphosphate (IPP) and its isomer dimethylallyl diphosphate (DMAPP). In plants, IPP and DMAPP are synthesized via two independent pathways: the mevalonic acid (MVA) pathway in the cytosol [1] and the 2-C-methyl-D-erythritol 4-phosphate (MEP) pathway in the plastids [2]. IPP and DMAPP are subsequently used as substrates by distinct short-chain prenyl diphosphate synthases that catalyze the head-to-tail condensation of one molecule of DMAPP with one, two or three IPP units to produce geranyl diphosphate (GPP; C_10_), farnesyl diphosphate (FPP; C_15_) and geranylgeranyl diphosphate (GGPP; C_20_), respectively [3], [4]. Pathway specialized branches starting from these prenyl diphosphates lead ultimately to the production of the astonishing number of isoprenoid end products synthesized by plants. GPP serves as a precursor for monoterpenoids, GGPP is a precursor of diterpenoids, gibberellins, carotenoids and abscisic acid, side chains of chlorophyll, phylloquinone, plastoquinone and tocopherols, and geranylgeranylated proteins, and FPP serves as a precursor for sesquiterpenoids, sterols, brassinosteroids, triterpenoids, polyprenols, side chains of ubiquinone, and farnesylated proteins [5]. It is generally accepted that the intracellular levels of these prenyl diphosphates and their precursors IPP and DMAPP must be strictly controlled to avoid deleterious effects on the metabolic flux through the pathway branches competing for these intermediates [6]. Indeed, overexpression of FPP synthase (FPS) in Arabidopsis [7], [8], [9] and phytoene synthase in tomato [10] results in altered levels of specialized isoprenoid end products that negatively affect plant performance.

Plants contain small FPS (EC 2.5.1.1/EC 2.5.1.10) isozyme families [11], [12], [13], [14], [15], [16], [17], [18]. This, together with the key position of FPP at a node of the isoprenoid pathway to which many branches leading to mitochondrial and cytosolic isoprenoids are connected [19], [20], has fuelled interest in deciphering the role of individual FPS isozymes in the isoprenoid pathway. Arabidopsis contains two genes, *FPS1* (At5g47770) and *FPS2* (At4g17190), encoding three FPS isozymes: FPS1L, FPS1S and FPS2. The *FPS1* gene encodes FPS1S and FPS1L, which differ only by an N-terminal extension of 41 amino acid residues that targets FPS1L into mitochondria [10], [13] whereas the *FPS2* gene encodes FPS2 that shares 90.6% amino acid identity with FPS1 isozymes [12]. Although the intracellular localization of Arabidopsis FPS1S and FPS2 remains to be experimentally established, both isozymes are predicted to localize in the cytosol since they do not bear any obvious subcellular targeting signal [12]. However, the possibility that one or both Arabidopsis short FPS isozymes might reside in a different compartment cannot be excluded. In fact, a *Catharanthus roseus* short FPS lacking a canonical peroxisomal targeting sequence partially localizes to the peroxisomes of *C. roseus* cells [21]. This observation would support the hypothesis for a partial localization of the early steps of the plant isoprenoid pathway in peroxisomes [22], [23] as reported to occur in mammals [24].

Studies in transgenic Arabidopsis expressing chimeric *FPS::GUS* genes [25] and analysis of microarray expression data [26] have shown that *FPS* genes are expressed in all organs throughout plant development, albeit at greatly different levels. *FPS1* is widely expressed in all tissues throughout plant development whereas expression of *FPS2* is mainly concentrated in floral organs, seeds and the early stages of seedling development. Characterization of Arabidopsis *fps1* and *fps2* single knockout mutants demonstrated that a single functional *FPS* gene is enough to sustain normal plant growth and development, thereby indicating that *FPS1* and *FPS2* can almost fully complement each other. However, simultaneous knockout of both *FPS* genes is embryo-lethal and severely impairs male genetic transmission [26]. Thus, the small Arabidopsis *FPS* gene family seemingly constitutes a redundant two-locus genetic network in which as long as one gene functions, no noticeable loss of plant fitness occurs. Nevertheless, *FPS1* and *FPS2* functions are not completely redundant. FPS activity measurements and analysis of sterol and ubiquinone levels, the major cytosolic and mitochondrial FPP-derived isoprenoids, have shown that FPS1S has a major role during most of the plant life cycle, whereas FPS2 has a predominant role in seeds and during the early stages of seedling development. In fact, FPS2 is by far the major contributor to total FPS activity in mature seeds, though shortly after germination FPS1 replaces FPS2 as the major provider of FPS activity. Hence, lack of FPS2, but not of FPS1, leads to a marked reduction of sitosterol content in mature seeds concomitant to a positive feedback regulatory response of 3-hydroxy-3-methylglutaryl coenzyme A reductase (HMGR), the enzyme that catalyzes the main regulatory step in the MVA pathway and downstream isoprenoid pathways [27]. The elevated levels of HMGR activity become essential to sustaining a flux through the isoprenoid pathway that is high enough to produce sufficient sterols and likely other isoprenoids needed for normal seed viability, as revealed by the hypersensitivity of *fps2* mutant seeds to the HMGR inhibitor mevastatin [26]. Correct quantitative and qualitative sterol composition is essential for proper seed development and viability, not only because sterols have well established roles in maintaining membrane structure and function [28] and as precursors of brassinosteroids [29], but also because they are involved in signalling pathways that are essential for normal embryogenesis [30], [31].

Biochemical characterization of individual FPS isozymes can also greatly contribute to the understanding of their physiological functions. So far, studies on the biochemical properties of plant FPSs have been conducted in a very limited number of species using purified native [32], [33] and recombinant [18], [34] enzyme preparations and, to the best of our knowledge, a comparative biochemical analysis between individual members of FPS isozyme families has only been reported in *Artemisia tridentata.* This plant contains two FPS isozymes that share 83% of amino acid identity. In spite of this, FDS-1 and FDS-2 exhibit different functional properties, which led the authors to propose specific cellular functions for each of the two isozymes [18]. In the present study, we report the results of a detailed functional and structural characterization of the two Arabidopsis short FPS isozymes FPS1S and FPS2. We also expand our previous *FPS* gene expression analysis [25] by establishing the spatial and temporal pattern of expression of the *FPS* genes during seed development, and present the results of functional complementation studies of the *fps2* single knockout mutant phenotypes [26] with chimeric *FPS* gene constructs. Altogether, findings reported herein provide new clues to understand the biological role of FPS isozymes in Arabidopsis.

## Materials and Methods

### Chemicals

Unlabelled IPP, DMAPP, GPP and FPP were purchased from Echelon Biosciences and [4-^14^C]IPP (60 mCi/mmol) was from GE Healthcare Life Sciences. Mevastatin (Calbiochem, www.merck-chemicals. com) was dissolved in EtOH to prepare a 5 mM stock solution.

### Plant Material and Growth Conditions

*Arabidopsis thaliana* wild-type (ecotype Col-3 qrt1/qrt1) and *fps2* mutant plants were grown *in vitro* on Murashige and Skoog (MS) medium (Duchefa, http://www.duchefa.com) solidified using 0.8% w/v agar, or *in vivo* on soil in a climate-controlled growth chamber, under long-day conditions (16 h light/8 h dark) at 22°C. When required, MS medium was supplemented with 1 µM mevastatin.

### Heterologous Expression of GST-FPS1S and GST-FPS2 in *E. coli* and Recombinant Protein Purification

Arabidopsis FPS proteins were expressed as N-terminal GST fusion proteins using a modified version of pGEX-3X plasmid (Pharmacia Biotech). The polylinker of pGEX-3X was modified by introducing a *Not*I restriction site upstream from the *Bam*HI site that allows to obtain native proteins having an N-terminal end without extra residues after cleavage with Factor Xa protease [35]. The cDNAs coding for FPS1S and FPS2 were amplified by PCR using, respectively, the forward primers 5′-**ATG**GAGACCGATCTCAAGTCAACC-3′ and 5′-**ATG**GCGGATCTGAAATCAACC-3′, the common reverse primer 5′-CGCGGATCC **CTA**CTTCTGCCTCTTGTAG-3′ (translation start and stop codons are shown in bold, and *Bam*HI restriction site is underlined) and plasmids pcNC3 [13] and pcNC2 [12] as templates, respectively. The resulting PCR products were digested with *Bam*HI, phosphorylated with T4 Polynucleotide kinase (Promega) and cloned into the *Not*I (blunt ended by nuclease S1 treatment) and *Bam*HI restriction sites of the modified pGEX-3X, yielding plasmids pGEX-3X-NotI-FPS1 and pGEX-3X-NotI-FPS2. These expression plasmids were transformed into the *E. coli* strain BL21 (DE3), harbouring pUBS520 encoding *E.coli* tRNA^Arg^ AGA/AGG [36], and transformed cells were grown overnight on LB plates supplemented with ampicillin (100 µg/mL). Plasmids were isolated from overnight cultures and their sequences were verified. To express the recombinant proteins, 30 mL of LB medium supplemented with ampicillin (100 µg/mL) and kanamycin (50 µg/mL) was inoculated with 0.5 mL of an overnight culture of BL21 (DE3) cells harbouring either pGEX-3X-NotI-FPS1 or pGEX-3X-NotI-FPS2 and grown at 37°C to an OD_600_ of 0.5–0.6. After induction with 0.4 mM isopropyl thio-β-D-galactopyranoside (IPTG) the cultures were shifted to 20°C and incubated for 16 additional hours at 200 rpm. *E.coli* cells were collected by centrifugation at 7,000 *g* for 5 min at 4°C, resuspended in 3 mL of PBS (80 mM Na_2_HPO_4_, 20 mM NaH_2_PO_4_, 100 mM NaCl, pH 7.5) and disrupted by sonication (0.5 min/mL suspension) while being chilled in a −10°C bath. Cell debris was removed by centrifugation at 15,000 *g* for 30 min at 4°C, and the resulting supernatant was loaded onto a 1 mL Glutathione-Sepharose 4B affinity chromatography column (GE Healthcare) pre-equilibrated with PBS. All procedures for enzyme purification were performed at 4°C unless otherwise indicated. The column was washed with a minimum volume of 10 mL of PBS and re-equilibrated with Factor Xa cleavage buffer (50 mM Tris-HCl, pH 7.5, 50 mM NaCl, 1 mM CaCl_2_). GST-FPS fusion proteins were digested by adding to the column 40U of Factor Xa (Amersham Biosciences) in cleavage buffer to the column. After overnight incubation at 20–22°C the resulting native FPS proteins were eluted with cleavage buffer. Fractions of 250 µL were collected and analyzed by 12,5% SDS-PAGE [37] after a quick estimation of protein concentration with a NanoDrop ND-1000 spectrophotometer (Thermo Fisher Scientific Inc.). Fractions enriched in FPS protein were pooled and the remaining Factor Xa protease was removed by treatment with Factor Xa removal resin (Qiagen) according to the manufacturer’s instructions. Glycerol was then added to a final concentration of 15% (v/v) and samples were stored frozen at −80°C. The purity of each FPS preparation was checked by SDS-PAGE. Protein concentration was determined by the method of Lowry [38] using BSA as a standard.

### FPS Enzyme Activity Assay and Kinetic Studies

FPS activity of purified recombinant FPS isozymes was measured in a total volume of 100 µL containing 30 mM PIPES (pH 7.0), 6 mM NaCl, 4 mM MgCl_2_, 150 mM sucrose, 10 µg/mL aprotinin, 2 µg/mL E64, 1 µg/mL pepstatin, 0.35 mM PMSF, 0.30 mg/mL bovine serum albumin (BSA), 100 µM [4-^14^C]IPP (6.97 µCi/µmol), 100 µM GPP and the appropriate amount of enzyme (between 10 and 40 ng). The reaction was initiated by the addition of the substrates after preincubation of the enzyme for 5 min at 37°C. The reaction was carried out at 37°C for 15 min and terminated by the addition of 585 µl of 2 M HCl pre-chilled at 0°C. Solid NaCl was added to saturation and the reaction products were acid hydrolysed by incubation for 30 min at 37°C. The mixture was extracted with 1 mL of n-hexane and the radioactivity in 500 µL of the hexanic phase was quantified by liquid scintillation counting. To measure FPS activity in extracts from plant tissues, shoots (between 200 and 250 mg) or seeds (between 25 and 40 mg) were mixed with extraction buffer (2 µL per mg of seedlings and 20 µL per mg of seeds) containing 50 mM PIPES, pH 7.0, 250 mM sucrose, 10 mM NaCl, 5 mM MgCl_2_, 5 mM DTT, 15 µg/mL aprotinin, 3 µg/mL E64, 1.5 µg/mL pepstatin, and 0.5 mM PMSF pre-chilled at 4°C and ground to a fine powder with mortar and pestle. The slurry was centrifuged at 200 *g* for 10 min at 4°C to remove cell debris and the resulting supernatant was collected and centrifuged again at 16,000 *g* for 20 min at 4°C. The supernatant was recovered and FPS activity (between 100 and 200 µg of protein) was assayed for 30 min at 37°C as described above. One unit of FPS is defined as the amount of enzyme that incorporates one nanomol of IPP into acid-labile products per minute and mg of protein at 37°C.

For pH dependence analysis, enzyme activity assays were carried out using MES (5.5, 6.0), PIPES (6.5, 7.0) and Tris-HCl (7.5, 8.0, 8.5, 9.0). The apparent *K*_m_ values for the substrates IPP, DMAPP and GPP were calculated from Lineweaver-Burk plots of FPS activity. The *K*_m_ values for DMAPP and GPP were determined with substrate concentrations in the range of 6.25 to 200 µM with a fixed IPP concentration of 100 µM. The *K*_m_ value for IPP was determined with concentrations in the range between 1 to 100 µM and a fixed DMAPP concentration of 200 µM. A nonlinear regression analysis in Sigma Plot 7.0 was used to calculate the kinetic parameters.

### Determination of HMGR Enzyme Activity and Sitosterol Content in Seeds

For HMGR activity measurements, seeds (50 mg) were frozen in liquid nitrogen, ground to a fine powder with mortar and pestle, and mixed with 0.4 mL of pre-chilled extraction buffer (40 mM sodium phosphate, pH 7.5, 100 mM sucrose, 30 mM EDTA, 50 mM NaCl, 10 mM DTT, 10 µg/mL aprotinin, 1 µg/mL E64, 0.5 µg/mL leupeptin, 1 µg/mL pepstatin, 0.5 mM PMSF and 0.25% (w/v) Triton X-100). The slurry was centrifuged at 200 *g* for 10 min at 4°C to remove cell debris and HMGR activity was immediately measured in the supernatant as previously described [39]. One unit of HMGR activity is defined as the amount of enzyme that converts one picomol of 3-hydroxy-3-methylglutaryl coenzyme A into MVA per min and mg of protein at 37°C. Sitosterol levels in seeds were analyzed by GC-MS as previously described [26].

### Western Blot Analysis

Aliquots (40–50 µg of protein) of the same seed extracts used for FPS activity measurements (16,000 *g* supernatant) were fractionated by 10% SDS-PAGE and electrotransferred onto Hybond-P polyvinylidene difluoride membranes (Amersham, Buckinghamshire, UK) at a constant intensity of 125 mA for 3 h at 4°C. The membrane was blocked in PBS pH 7.5, 0.5% (v/v) Tween 20 and 5% (v/v) Blotto non-fat dry milk (Santa Cruz Biotechnology inc.) for 16 h at 4°C, and incubated with rabbit polyclonal anti-FPS1S antibody [8] (1∶8000 dilution in blocking solution) for 1 h at room temperature. The membrane was then incubated with goat anti-rabbit IgG conjugated to peroxidase (Amersham) (1∶60000 dilution in blocking solution) for 1 h at room temperature. The FPS1S-antibody complex was visualized using the ECL Advance Western blotting system (GE Healthcare) according to the manufacturer's instructions. Protein loading was assessed by Coomassie blue staining of the membranes.

### Differential Scanning Fluorimetry

The difference in thermal stability between FPS1S and FPS2 was analyzed by differential scanning fluorimetry (DSF) [40]. In brief, 20 µL reactions were set up on a 96-well thin-wall plate (Bio-Rad) containing 3–14 µM each protein in assay buffer (50 mM Tris-HCl, pH 7.5, 50 mM NaCl, 1 mM CaCl_2_, 15% (v/v) glycerol and 5× Sypro Orange (Invitrogen). Assay buffer was added instead of protein in the control samples. The plates were sealed with optical-quality sealing tape (Bio-Rad) and heated on a iQ5 Real Time (RT)-PCR instrument (Bio-Rad) from 20–80°C in increments of 0.2°C. Fluorescence was monitored with a charge-coupled device (CCD) camera using 490 and 575 nm as emission and excitation wavelengths, respectively. The mid-point temperature of the unfolding transition or melting temperature (*T*_m_) was calculated by fitting a Boltzmann model to the fluorescence imaging data after eliminating data beyond the fluorescence intensity maximum.

### Homology Modelling

To construct homology models of Arabidopsis FPS1S and FPS2 that could be directly comparable with respect to their sequence differences, we chose as template the crystal structure of unliganded human FPS (PDB 2F7M) [41]. The sequence identity of human FPS and FPS1S was 45% over 99% of its length (339 out of 343 amino acids) and between human FPS and FPS2 was 46% over 99% of its length (338 out of 342 amino acids). The phosphate ion and the four water molecules found in the active site of FPS in this structure were kept to maintain a stable conformation of the active site loops during modelling. First, we threaded the correct FPS1S and FPS2 sequences onto the template structure using Modeller 9.10 [42] and then selected the 10 best models out of 1000 independent models on the basis of the Z-DOPE normalized scores, which ranged from –1.916 to –1.337 for FPS1S and from –1.949 to –1.529 for FPS2 (a Z-DOPE of less than –1 indicates a plausible model with 80% of the Cα atoms lying within 3.5 Å of their correct positions). Next, the top ten models for each protein were refined and minimized using the Rosetta force field and Monte Carlo sampling methods [43], [44] and the improved models were clustered and analyzed to obtain a final model. The final models for FPS1S and FPS2 showed correct stereochemistry as assessed by MolProbity [45].

### In Silico Evaluation of Free Energy and Structure Changes Upon Mutation

Free energy (*DD*G) and structure changes upon mutation were calculated using two established methods, the Rosetta *DD*G application [46] and the publicly available CC/PBSA web server (http://ccpbsa.biologie.uni-erlangen.de/ccpbsa/) [47]. The changes in stability predicted by the two independent calculations were analyzed in the context of the predicted structural changes. The Rosetta *DD*G protocol corresponds to row 16 of a recent benchmarking study [46]. Row 16 protocol first repacks all residues according to Rosetta standard sidechain sampling procedures while keeping the backbone fixed, and then minimizes all backbone and sidechain degrees of freedom. Energies are calculated for 50 wild-type and mutant sequence contexts and the predicted *DD*G is the difference in the free energy between the mutant and wild-type protein. The CC/PBSA method is accessed via a web interface where the coordinates are submitted along with a string describing the desired mutation. In a nutshell, the CC/PBSA method generates two random conformational ensembles each one of them consisting of 300 wild-type and mutant minimized structures for which energies are evaluated using a custom energy function. Those energy contributions are scaled to reproduce experimentally measured free energies using five-fold cross validation.

### Gene Constructs for Mutant Complementation

To construct plasmid pCAM-FPS2p::FPS1S, a 1388-bp fragment including 1329 bp of the *FPS2* gene promoter, the 5′ untranslated region and the ATG translation start codon, was amplified by PCR using genomic DNA as a template, forward primer 5′-GCGTCGACAGCTTGGAGCATAAGAAG-3′ and reverse primer 5′-TCCGCCAT**G**GATAGGATCAAGG-3′. A *Sal*I restriction site was added at the 5′ end of forward primer and an *Nco*I site encompassing the ATG start codon of FPS2 was created in the reverse primer by introducing a G (shown in bold) instead of a C. A 2353-bp fragment including the region encoding FPS1S and 339 bp of the 3′-non coding region was amplified by PCR using genomic DNA as a template, forward primer 5′-AGCTCTTC**C**ATGGAGACCGATC-3′ and reverse primer 5′-TTGGAGCTCTTTGGAATGGAATGTAGG-3′. An *Nco*I restriction site encompassing the ATG start codon of FPS1S was created by introducing a C (shown in bold) instead of a G in the forward primer and a *Sac*I restriction site was added at the 5′ end of reverse primer. Both genomic fragments were cloned into pGEM-T Easy vector (Promega), excised by digestion with either *Sal*I and *Nco*I or *Nco*I and *Sac*I, and cloned into the *Sal*I and *Sac*I sites of pBluescript KS^+^ in a three-piece ligation yielding pBFPS2p::FPS1S. The entire FPS2p::FPS1S fragment was then excised by digestion with *Sal*I and *Sac*I and cloned into pCAMBIA2300 yielding plasmid pCAM-FPS2p::FPS1S.

To create plasmid pCAM-FPS2p::FPS1S-mutdis, a 1375-bp fragment including 1329 bp of the *FPS2* gene promoter was amplified by PCR using genomic DNA as a template, forward primer 5′-GCGTCGACAGCTTGGAGCATAAGAAG-3′ and reverse primer 5′-CCCAAGCTTGATAGGATCAAGGAAGGTGT-3′. Restriction sites for *Sal*I and *Hind*III (underlined) were added at the 5′ end of forward and reverse primers, respectively. The amplified fragment was cloned into pGEM-T Easy vector yielding pGEM-FPS2p. A 2472-bp fragment including the entire coding region of the *FPS1* gene and 339 bp of the 3′-non coding region was amplified by PCR using genomic DNA as a template, forward primer 5′-GGGATAT**C**AGTGTGAGTTGTTGTTGT-3′ and reverse primer 5′-TTGGAGCTCTTTGGAATGGAATGTAGG-3′. Restriction sites for *Eco*RV and *Sac*I (underlined) were added at the 5′ end of forward and reverse primers, respectively. In the forward primer the third base of the ATG codon corresponding to the translation start codon of FPS1L isoform was changed to C (shown in bold). The amplified fragment was cloned into the *Eco*RV and *Sac*I sites of pBluescript SK^+^ yielding pBFPS1S-mutdis. The *FPS1* gene fragment was then excised from pBFPS1S-mutdis with *Eco*RV and *Sac*I and cloned into plasmid pGEM-FPS2p, which had been previously digested with *Hind*III, treated with nuclease S1 to produce blunt ends and digested again with *Sac*I (in the pGEM-T polylinker) yielding pGEM-FPS2p::FPS1S-mutdis. The entire FPS2p::FPS1S-mutdis fragment was then excised by digestion with *Sal*I and *Sac*I and cloned into pCAMBIA2300 yielding plasmid pCAM-FPS2p::FPS1S-mutdis.

To construct plasmid pCAM-FPS1p::FPS2, a 1526-bp fragment including 1338 bp of the *FPS1* gene promoter and 185 bp of the 5′ leader region up to the ATG translation start codon of FPS1S was amplified by PCR using DNA from genomic clone pgNC241 as a template [12], forward primer 5′-GCGTCGACATAGTAGTTAATGTTGGGG-3′ and reverse primer 5′-TCTCCAT**G**GAAGAGCTTTGGATACG-3′. A *Sal*I site was added at the 5′ end of forward primer and an *Nco*I site encompassing the ATG start codon of FPS1S was created by introducing a G (shown in bold) instead of a T in the reverse primer. A 2553-bp fragment including the entire coding region of the *FPS2* gene and 438 bp of the 3′-non coding region was amplified by PCR using genomic DNA as a template, forward primer 5′-GATCCTATC**C**ATGGCGGATCTG-3′ and reverse primer 5′-AGCGAGCTCATTTCCACTAATCTTCTCG-3′. An *Nco*I restriction site encompassing the ATG start codon of FPS2 was created by introducing a C (shown in bold) instead of an A in the forward primer. A *Sac*I restriction site was added at the 5′ end of reverse primer. Both genomic fragments were cloned into pGEM-T Easy vector, excised by digestion with either *Sal*I and *Nco*I or *Nco*I and *Sac*I, and cloned into the *Sal*I and *Sac*I sites of pBluescript KS^+^ in a three-piece ligation yielding pBFPS1p::FPS2. The entire FPS1p::FPS2 fragment was then excised by digestion with *Sal*I and *Sac*I and cloned into pCAMBIA1300-T-Nos yielding plasmid pCAM-FPS1p::FPS2. Plasmid pCAMBIA1300-T-Nos was generated by introducing the T-Nos sequence from pBI221 into the *Sac*I and *Eco*RI sites of pCAMBIA1300.

To create plasmid pCAM-FPS1mutdisp::FPS2, a 1526-bp fragment of the *FPS1* flanking region was amplified by PCR using as a template a chimeric translational *FPS1S::GUS* gene fusion in which the ATG start codon of FPS1L had been converted to an ATC codon by site directed mutagenesis [25] and the same forward and reverse primers used to construct pCAM-FPS1p::FPS2. The amplified fragment was cloned into pGEM-T Easy yielding pGEM-FPS1mutdisp. The *FPS1* gene fragment was then excised with *Sal*I and *Nco*I and cloned into pBFPS1p::FPS2 to replace the corresponding non-mutated region of *FPS1* promoter, yielding plasmid pBFPS1mutdisp::FPS2. The entire pBFPS1mutdisp::FPS2 fragment was then excised with *Sal*I and *Sac*I and cloned into pCAMBIA1300-T-Nos yielding plasmid pCAM-FPS1mutdisp::FPS2.

All PCR fragments used to construct plasmids described above were sequenced to exclude amplification artifacts. The correct fusion of the genomic fragments was also confirmed by sequencing.

### Plant Transformation and Transgene Expression Analysis

*Agrobacterium tumefaciens* strain GV3101 harbouring plasmids described above was used to transform Arabidopsis *fps2-1* mutant plants by the floral dip method [48]. Seeds from infiltrated plants were surface sterilized and sown in Petri dishes containing solid MS medium supplemented with 50 µg/mL kanamycin. Antibiotic-resistant seedlings (T_1_) were transplanted into soil and grown to maturity. Lines homozygous for the transgenes containing a single insertion were selected by segregation analysis of the kanamycin resistance trait.

To analyze the expression of the transgenes introduced into the *fps2-1* mutant, total RNA was isolated from 12-days-old seedlings form *fps2-1* lines harbouring *FPS2p::FPS1S*, *FPS2p::FPS1-mutdis*, *FPS1p::FPS2*, or *FPS1mutdisp::FPS2*. Total RNA (1,5 µg) was treated with DNAse (Ambion) and single-stranded cDNA pools were synthesized using oligo-dT primer and SuperScript III reverse transcriptase (Invitrogen) according to standard protocols. PCR reactions were carried out by 35 cycles of amplification (45 s at 94°C, 60 s at 50°C and 90 s at 72°C with a 5 min final extension at 72°C) using as template 7.5 µL of a 1∶10 dilution of the corresponding single-stranded cDNA pools and 1 unit of Taq polymerase (Biotools). Primers 5′- GGCTTTGCACACCTTCCTTG-3′ and 5′-CCTGTGGATGTGATTGCGAAG-3′ were used for expression analysis of *FPS2p::FPS1S* and *FPS2p::FPS1-mutdis* genes, and primers 5′-GGTGGGAGTCTCTATCGTCGTCGTATCCAA-3′ and 5′-CGGAGAGGCCCGAGTATG-3′ were used for expression analysis of *FPS1p::FPS2* and *FPS1mutdisp::FPS2* genes. The expression of the *ACT2* (At3g18780) gene was analyzed using primers 5′-GATCTGGCATCACACTTTCTAC-3′ and 5′- GCCTTGGAGATCCACATCTGCTG-3′. The expression of the *PP2AA3* (At1g13320) gene encoding the 65 kDa regulatory subunit of protein phosphatase 2A (PP2A) was analyzed using primers 5′-TAACGTGGCCAAAATGATGC-3′ and 5′-GAAGCCAACATTAACATTAGTAGC-3′.

### GUS Assay in *Arabidopsis* Seeds

Siliques were harvested, opened longitudinally, placed in GUS assay buffer (50 mM phosphate buffer, pH 7.0, 0.2% (v/v) Triton X-100, 20 mM X-Gluc and 2 mM potassium ferricyanide) and subjected to vacuum for 10 min. After incubation for 24 hours at 37°C, siliques were placed in ethanol:acetic acid (1∶1) and incubated for either 4 hours (young seeds with embryos at the globular and heart stages) or 8 hours (mature seeds with embryos at torpedo and cotyledon stages). GUS-stained seeds were cleared in Hoyer’s medium for 3–4 days in darkness [49]. Dissected seeds were placed on a slide covered with a coverslip, stored in darkness for 24 hours and observed under a Zeiss Axiophot microscope equipped with Nomarski optics. Photographs were taken using the same microscope equipped with an Olympus DP70 photo camera.

### Expression of GFP-FPS1S, GFP-FPS2, FPS1S-GFP and FPS2-GFP in Agroinfiltrated Leaves of *N. benthamiana* Plants

The FPS1S coding sequence (1029 bp) was amplified by PCR using forward primer 5′-ACGCGTCGACA**ATG**GAGACCGATCTCAAGTCAACC-3′, reverse primer 5′-CTGTCGATATCCCTTCTGCCTCTTGTAGATCTTAGC-3′ and plasmid pcNC3 [13] as a template. The sequence coding for FPS2 (1039 bp) was amplified using forward primer 5′-ACGCGTCGACA**ATG**GCGGATCTGAAATCAACCTTC-3′, reverse primer 5′-GAGTATGATATCCCTTCTGCCTCTTGTAGATCTTAGC-3′ and plasmid pcNC2 [12] as a template. Translational start codons are shown in bold. *Sal*I and *Eco*RV restriction sites (underlined) were added at the 5′ end of the forward and reverse primers, respectively. The amplified fragments were cloned into the corresponding sites of plasmid pENTR3C (Gateway®, Invitrogen) yielding plasmids pENTR-FPS1S and pENTR-FPS2. Both cDNA fragments were subsequently transferred from pENTR-FPS1S and pENTR-FPS2 to pMDC43 and pMDC83 (Gateway®, Invitrogen) yielding plasmids pMDC43FPS1S, pMDC43FPS2, pMDC83FPS1S and pMDC83FPS2, in which the FPS coding sequences were fused in-frame to the 5′- or 3′-ends of the green fluorescent protein (GFP) coding sequence. In all cases the coding sequences were under the transcriptional control of the CaMV35S gene promoter. All constructs were sequenced to confirm the in-frame fusions. Plasmids coding for the different protein fusions were transformed into *Agrobacterium tumefaciens* strain EHA105. The recombinant *A. tumefaciens* strains were grown overnight at 28°C in YEB liquid medium supplemented with 100 µg/mL rifampicine and 25 µg/mL kanamycin. Cells were harvested by centrifugation and resuspended to an OD_600_ of 0.150 in a solution containing 10 mM MgCl_2_, 10 mM HEPES, pH 5.6, and 200 µM acetosyringone (3,5-dimethoxy-4′-hydroxy-acetophenone). Prior infiltration, bacterial suspensions were incubated at room temperature for 3 h. For co-expression experiments, suspensions of *A. tumefaciens* harbouring the FPS-GFP expression constructs were mixed with *A. tumefaciens* cultures harbouring constructs for expression of the cyan fluorescent protein (CFP)-peroxisome marker (CFP-SKL) [50] and the tobacco etch polytovirus helper component protein (HC-Pro) silencing suppressor [51] in a 1∶1:1 ratio. Leaves of 2–4 week old *N. benthamiana* plants were infiltrated by gently appresing a 2-ml syringe without a needle to the abaxial surface of fully expanded leaves. Following a 2–3 day incubation of infiltrated plants under long-day conditions at 25°C and 60% humidity, abaxial epidermis of leaf tissue was examined by confocal laser-scanning microscopy using a Leica SP5 microscope (Leica Microsystems). GFP and CFP were excited by using 488 and 458 laser lines, respectively. Images were acquired sequentially to avoid crosstalk between channels. LAS-AF Lite 2.6.0 software was used for image capture and for merging false-coloured images of transiently co-transformed cells.

## Results

### Biochemical Characterization of Recombinant FPS1S and FPS2 Isozymes

**Figure 1:**
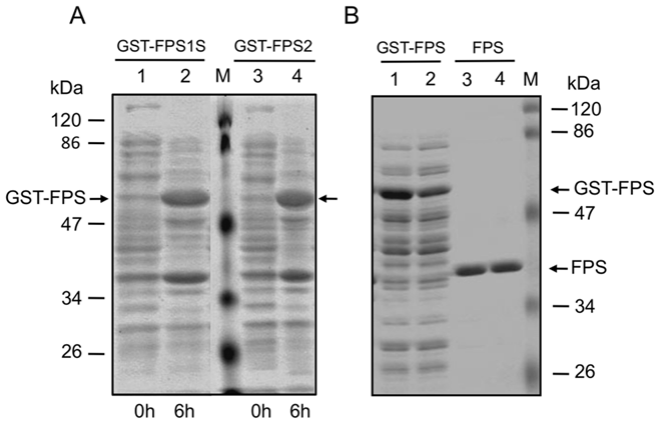
Expression in *E. coli* and purification of recombinant FPS1S and FPS2 proteins. (A) Total protein extracts from *E. coli* cells harbouring either pGEX-3X-NotI-FPS1 or pGEX-3X-NotI-FPS2 before (lanes 1 and 3) and after induction (lanes 2 and 4) of GST-FPS1S and GST-FPS2 expression with 0.4 mM IPTG for 6 hours at 22°C. (B) Soluble protein extracts of IPTG-induced *E. coli* cells harbouring either pGEX-3X-NotI-FPS1 (lane 1) or pGEX-3X-NotI-FPS2 (lane 2), and purified native FPS1S (lane 3) and FPS2 (lane 4) protein preparations after Glutathione-Sepharose 4B affinity column chromatography, proteolytic digestion with Factor Xa and protease removal. Arrows indicate the position of GST-FPS protein fusions and purified native FPS proteins. Molecular masses of standards (M) are indicated in kDa.

Arabidopsis FPS1S and FPS2 isozymes were expressed in *E. coli* as soluble N-terminal GST fusions (Figure 1A). The resulting recombinant proteins were purified through Glutathione-Sepharose 4B affinity chromatography, digested with Factor Xa protease to release native FPS1S and FPS2 enzymes, and further purified to remove both the excised GST moiety and Factor Xa protease. SDS-PAGE analysis showed that this purification procedure yielded highly purified preparations of both enzymes (Figure 1B) that were used for biochemical characterization.

**Figure 2:**
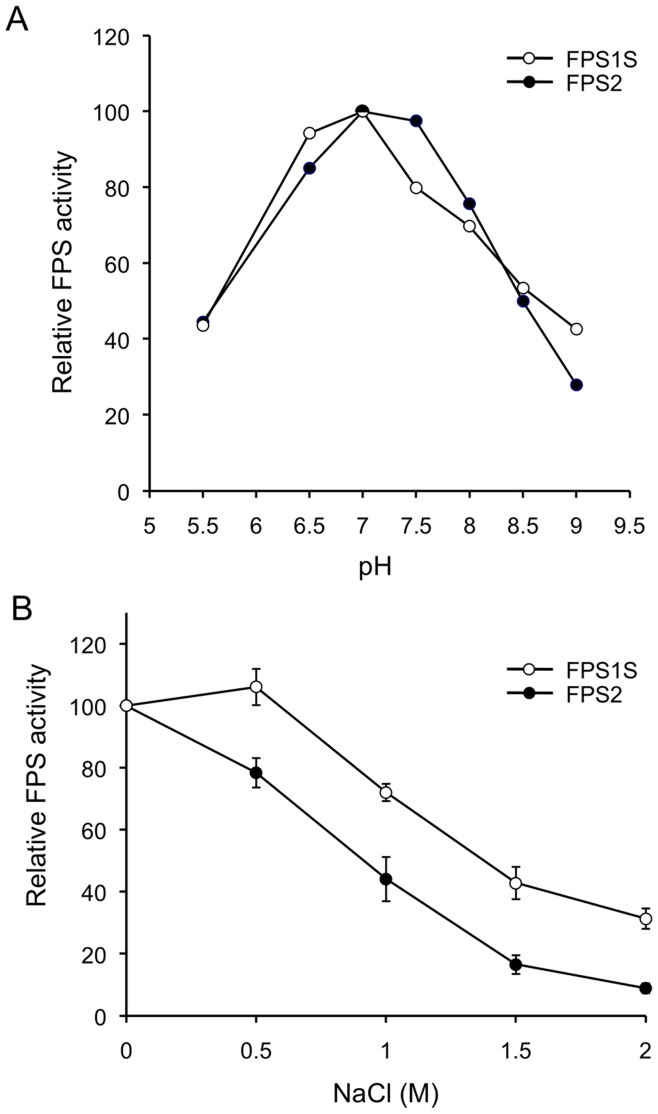
Effect of pH and NaCl on FPS1S and FPS2 enzyme activity. (A) FPS activity of purified FPS1S and FPS2 was determined at pH values ranging from 5.5 to 9.0 as described under Materials and Methods. Enzyme activities are expressed relative to the maximal activity values for FPS1S and FPS2. (B) Effect of NaCl on FPS1S and FPS2 enzyme activity. FPS activity of purified FPS1S and FPS2 was determined at the indicated NaCl concentrations. Enzyme activities are expressed relative to the activity values for FPS1S and FPS2 measured without NaCl. The mean values and SE were calculated from three independent experiments.

Prenyltransferases are known to require a divalent metal ion for catalytic activity. Thus, we first analyzed the effect of different Mg^2+^ concentrations on FPS1S and FPS2 activity. Similar values of activity were obtained when the enzyme activity was measured in the presence of MgCl_2_ concentrations ranging between 1 and 5 mM (data not shown). The optimal pH range for FPS1S and FPS2 was also determined. Both enzymes showed a similar pH-rate profile with only minor differences (Figure 2A). Maximal activity for both enzymes was observed at a pH value of 7.0, albeit the pH-rate profile of FPS1S was slightly shifted toward more acidic pH values compared to FPS2. We next investigated the effect of NaCl on the activity of FPS1S and FPS2 using concentrations in the range from 0 to 2 M. As shown in Figure 2B, the activity of both enzymes progressively declined as the concentration of NaCl increased, though in the case of FPS1S this effect was observed only at NaCl concentrations higher than 0.5 M. At all NaCl concentrations tested, FPS2 was markedly more sensitive to the inhibitory effect of NaCl than FPS1S. Purified FPS1S and FPS2 were also subjected to kinetic analyses and the resulting steady-state kinetic constants are shown in Table 1. Both enzymes displayed typical Michaelis-Menten behaviour, as observed for other FPSs, and exhibited similar *K*_m_ values in the µmolar range (from 8.3 to 31.5 µM) for both IPP and the allylic substrates DMAPP and GPP. The affinity (*K*_m_) of FPS1S and FPS2 for the reaction intermediate GPP was approximately 2.7-fold higher than for DMAPP, thus indicating a preference of both enzymes for the allylic intermediate. Similarly, comparison of the specificity constants (k_cat_/*K*_m_) indicated a 2-fold higher catalytic efficiency for GPP compared to DMAPP. The inhibitory effect of the reaction product FPP on FPS1S and FPS2 activity was analyzed using concentrations in the range from 0 to 1 mM. The activity inhibition profile was nearly identical for both enzymes, with a maximal 30% reduction of activity at the highest FPP concentration assayed.

**Table 1:** Steady-state kinetic constants for FPS1S and FPS2.

Variable substrate	Kinetic parameter	FPS1S	FPS2
IPP	*K*_m_ (μM)	23.1±0.4	31.3±0.5
	k_cat_ (min^−1^)	62.5	269.0
	k_cat_/*K*_m_ (min^−1^μM^−1^)	2.7	8.6
DMAPP	*K*_m_ (μM)	22.7±2.1	31.5±0.5
	k_cat_ (min^−1^)	52.2	197.3
	k_cat_/*K*_m_ (min^−1^μM^−1^)	2.3	6.2
GPP	*K*_m_ (μM)	8.3±1.2	11.3±1.0
	k_cat_ (min^−1^)	36.4	132.8
	k_cat_/*K*_m_ (min^−1^μM^−1^)	4.4	11.7

The *K*_m_ mean values and SE were calculated from three independent experiments.

k_cat_ values were calculated for the dimeric form of the enzyme.

**Figure 3:**
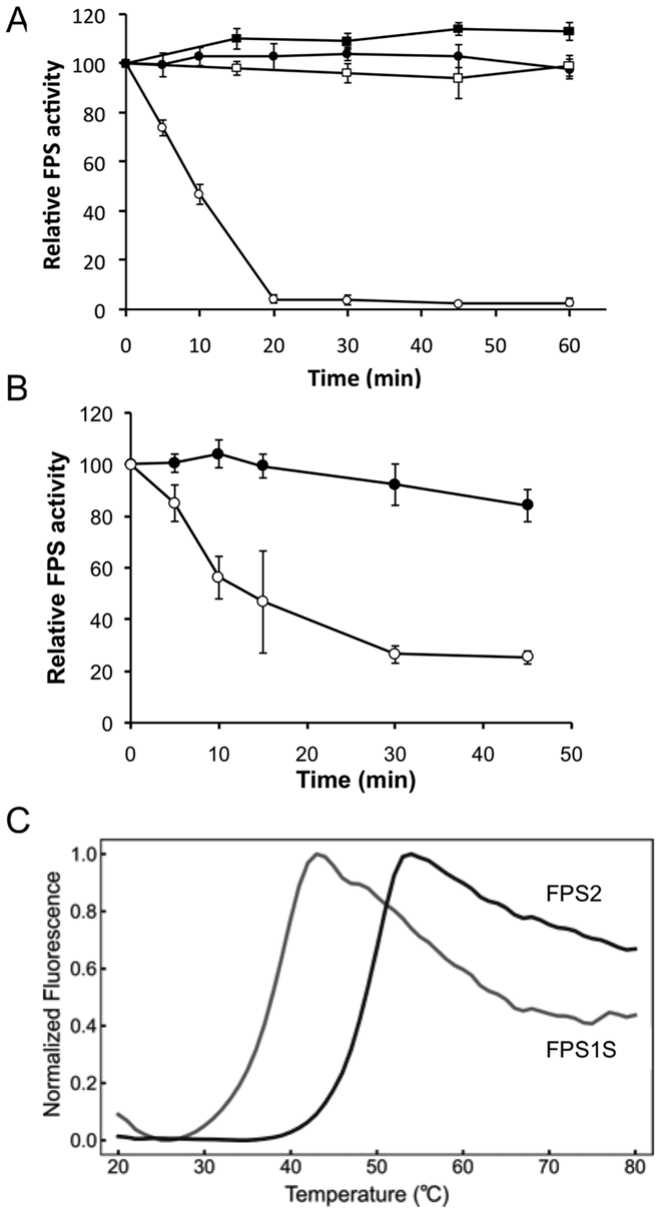
Thermal stability of FPS1S and FPS2 enzyme activity. (A) Activity of purified FPS1S (open symbols) and FPS2 (closed symbols) was measured after incubation at either 37°C (squares) or 45°C (circles) for the indicated time periods. (B) FPS activity in 16,000 *g* protein extracts from *fps1-1* (FPS2 activity, closed circles) and *fps2-1* (FPS1 activity, open circles) mutants was determined after incubation at 45°C for the indicated times. In both cases enzyme activities are expressed relative to the FPS activity values at time 0 min and the mean values and SE were calculated from three independent experiments. (C) Differential scanning fluorimetry (DSF) results plotted as change in fluorescence emission intensity (normalized to unity at its maximum) with increasing temperature (20–80°C). The FPS1S and FPS2 curves correspond to 6 µM enzyme.

Interestingly, FPS2 preparations consistently showed higher specific activity than the FPS1S ones. In fact, FPS2 showed catalytic rate constant (k_cat_) values for IPP, DMAPP, and GPP that were 3.2-, 2.7- and 2.7-fold higher, respectively, than FPS1S, thus indicating that FPS2 is a more efficient catalyst than FPS1S. These differences cannot be attributed to differential stability of the enzymes either under storage conditions or during the purification procedure since both retained more than 90% of their initial activity after 2 months of storage at −80°C and their activity remained virtually unaffected after incubation for 1 hour at 37°C (Figure 3A). In contrast to this later observation, we observed a markedly different response of FPS1S and FPS2 to treatment at 45°C for different time-periods. As shown in Figure 3A, FPS2 activity remained unaltered after incubation for 1 hour at 45°C whereas FPS1S activity was completely abolished after 20 minutes at the same temperature. A similar result was obtained when protein extracts from the Arabidopsis *fps1-1* and *fps2-1* single knockout mutants bearing only one functional *FPS* gene (*FPS2* and *FPS1* respectively) were incubated at 45°C for different time-periods and assayed for FPS activity. Again, incubation at 45°C had almost no effect on FPS2-derived activity in extracts from *fps1-1* plants, whereas FPS1-derived activity in extracts from *fps2-1* plants was severely diminished upon incubation at the same temperature (Figure 3B). These differences in thermal stability were corroborated by differential scanning fluorimetry (DSF) [40]. The melting temperature (*T*_m_) derived from the DSF data is a useful diagnostic tool because greater thermal stability is associated with a positive shift in *T*_m_ with respect to a reference *T*_0_ (*T*_m_ – *T*_0_ =  *DT*_m_ >0) and vice versa. DSF experiments with 3–14 µM FPS1S or FPS2 in FPS activity assay buffer yielded *T*_m_ values of 37°C and 48.3°C, respectively, a difference in *T*_m_ of 11.3°C (Figure 3C). Based on these results, it can be concluded that FPS2 is more resistant to thermal inactivation than FPS1S because FPS2 protein is thermodynamically more stable than FPS1S.

### Structural Basis of the Enhanced Thermal Stability of FPS2

**Figure 4:**
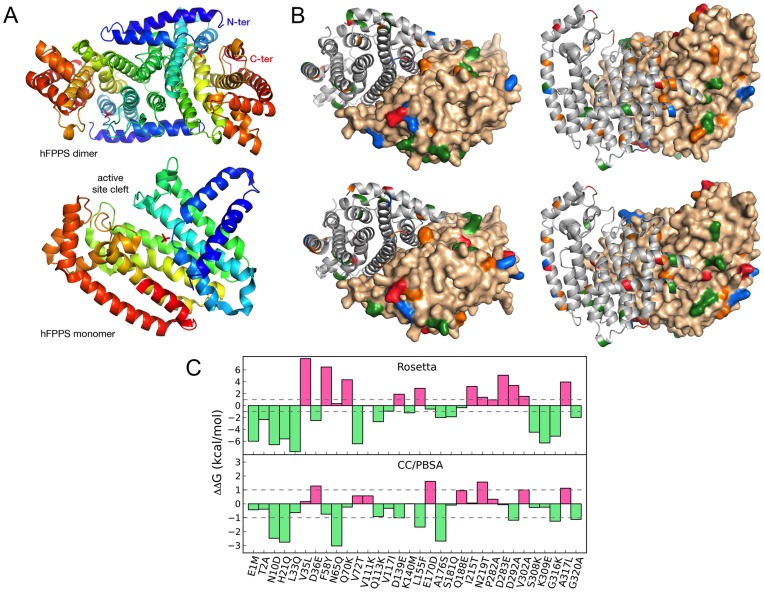
Homology modelling of FPS1S and FPS2 proteins and in silico *DD*G calculations. (A) Ribbon representation of the dimeric and monomeric structures of human FPS (PDB 2F7M), which was used to template the threading of Arabidopsis FPS1S and FPS2. The active site cleft is labelled and a bound phosphate ion is shown in sticks. (B) Sequence substitutions between FPS1S (top) and FPS2 (bottom) were mapped onto the ribbon structure (left monomer) or the molecular surface (right monomer) of the homology modeled dimers. Chemical character is colour coded as follows: red, acidic (Asp, Glu); blue, basic (Arg, Lys, His); green, polar (Ser, Thr, Asn, Gln, Tyr); orange, apolar (Met, Phe, Pro, Trp, Val, Leu, Ile, Ala). (C) Histogram of *DD*G (kcal/mol) upon single-site substitution calculated using Rosetta *DD*G application (top) or CC/PBSA (bottom). Mutations predicted to occur with a decrease in *DD*G are coloured green and those expected to increase *DD*G are coloured pink. Horizontal dashed lines at –1 to +1 kcal/mol bound the neutral area where *DD*G is supposed to contribute little to the overall stabilization or destabilization of the mutated protein.

To further our understanding of the differential thermal stability between FPS1S and FPS2, homology models for both proteins were built using as a template the crystal structure of unliganded human FPS (PDB 2F7M) [41] (Figure 4A) and carefully assessed with respect to their stereochemistry. Best models for both enzymes showed correct stereochemical parameters and native dimer interfaces using various stringent criteria [45], [52]. The root-mean-square (r.m.s.) deviation between each model and the template structure was 0.26 Å and between the two models was 0.29 Å. Mapping of the sequence substitutions between FPS1S and FPS2 onto their respective ribbon structures and molecular surfaces (Figure 4B) showed that the immense majority of these substitutions occur at the outer surface of the enzyme.

Detailed analysis of the thirty-two substitutions that separate FPS2 from FPS1S revealed that the increase in surface polarity and the greater capacity to form surface salt bridges of FPS2 compared with FPS1S appear to be determining factors of the enhanced thermostability of FPS2. Indeed, there are several discernable trends in amino acid substitution between FPS2 and FPS1S that lends support to this hypothesis. First, FPS2 has four charged residues more than FPS1S of which three are basic and one is acidic. Secondly, polar and charged sidechains in FPS2 tend to be longer on average than in FPS1S (e.g., substitutions follow Asp  = > Glu, Asn  = > Gln), which could afford greater rotameric freedom and thereby facilitate formation of polar interactions on the protein’s surface. In summary, inspecting the FPS1S and FPS2 homology models around each mutated residue suggested that as many as three additional salt bridges (and several strong hydrogen bonds) could be established in FPS2 compared to FPS1S. Other metrics of structure stability, such as sidechain hydrogen bonds (31 in all/4 strong versus 30 in all/2 strong) and salt bridges across the dimer interface (7 versus 5) interface did also favour FPS2 with respect to FPS1S. Interestingly, a number of aromatic-aromatic, aromatic-sulfur and pi-cation interactions seem to be more prevalent in FPS2 than in FPS1S, and could also make a sizable contribution to the overall thermal stability of the two enzymes.

To accurately calculate the changes in free energy and structure induced by single residue substitutions we applied the Rosetta *DD*G [46] and CC/PBSA [47] methods to predict the free energy and structure changes underwent by FPS1S as single FPS2-mimicking substitutions are introduced. Results indicated that the cumulative free energy change summed over all mutated residues was energetically very favourable regardless of the method used, in qualitative agreement with the results from DSF experiments. Moreover, the Rosetta *DD*G and CC/PBSA protocols flag 58% (15 versus 11) and 69% (9 versus 4) of the non-neutral substitutions as stabilizing (*DD*G < –1 kcal/mol). Of the non-stabilizing mutations, Rosetta *DD*G brands more mutations than CC/PBSA as destabilizing (*DD*G > +1 kcal/mol) than neutral (*DD*G between –1 and +1 kcal/mol). Figure 4C shows this pattern for both the Rosetta *DD*G and the CC/PBSA protocols. Of the 21 out of 32 substitutions that change any residue in FPS1S to a polar/charged residue in FPS2, 85% (18) are predicted as non-destabilizing (the three exceptions are Q70K, E170D and D283E) thereby providing corroborative evidence for the important role of surface electrostatic interactions for FPS2 thermal stability. Perhaps one of the larger discrepancies between the two prediction methods involves substitutions F58Y, N65Q, Q70K and V72T, for which *DD*G estimates are reversed. These four substitutions, which are located in three consecutive helices, cluster tightly together around a 6-Å sphere and therefore it is plausible that the true structure of FPS2 had undergone compensatory changes that cannot be accurately captured by our single-site mutation calculations. Future structural studies of FPS1S and FPS2 should provide more accurate rationales for the observed stability differences.

### Subcellular Localization of FPS1S and FPS2

**Figure 5:**
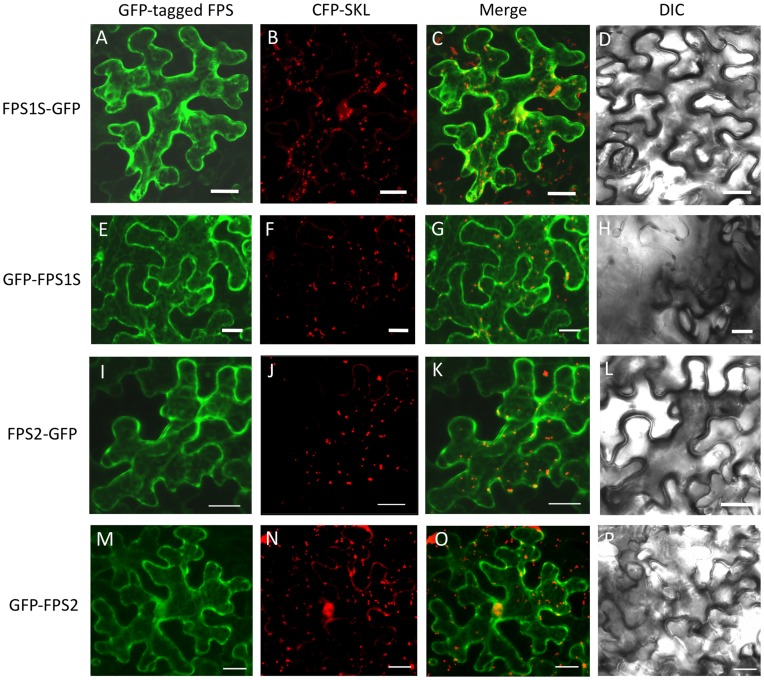
Subcellular localization of GFP-tagged FPS1S and FPS2 proteins. Confocal laser scanning micrographs showing the distribution of fluorescence in agroinfiltrated *N. benthamiana* epidermal cells transiently co-expressing FPS1S-GFP (A), GFP-FPS1S (E), FPS2-GFP (I) or GFP-FPS2 (M) with the peroxisome protein marker CFP-SKL (B, F, J and N). Co-localization evaluation of GFP-tagged FPS proteins with the peroxisome protein marker (C, G, K and O). Differential interference contrast (DIC) images showing the morphology of transformed cells (D, H, L and P). Scale bars = 20 µm.

Arabidopsis FPS1S and FPS2 isoforms have long been considered to localize in the cytosol, mainly because no obvious organellar targeting signals have been detected in their primary structure. To provide experimental data supporting this assumption, the subcellular localization of Arabidopsis FPS1S and FPS2 was investigated by transiently expressing N- and C-terminal fusions of both FPS isoforms to the GFP in agroinfiltrated *N. benthamiana* leaf cells (Figure 5). Confocal laser microscopy analysis of the transfected cells revealed that all four tested proteins (GFP-FPS1S, FPS1S-GFP, GFP-FPS2, and FPS2-GFP) showed a diffuse pattern of fluorescence throughout the cytosol that was completely different from the punctuate fluorescence pattern characteristic for peroxisomal proteins, as revealed by comparison with the fluorescence signal of the peroxisomal marker CFP-SKL. These results strongly suggested that both FPS1S and FPS2 localize in the cytosol and not in the peroxisomes.

### Expression Pattern of Arabidopsis *FPS1* and *FPS2* Genes during Seed Development

**Figure 6:**
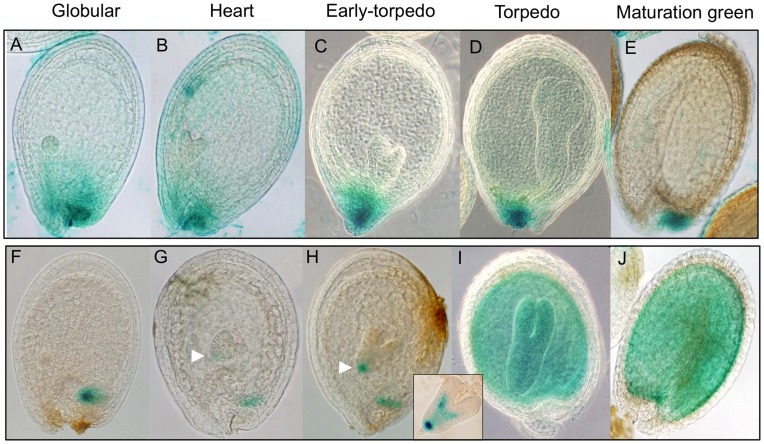
Histochemical analysis of GUS activity during seed development in Arabidopsis lines harbouring transgenes *FPS1S::GUS* (panels A to E) and *FPS2::GUS* (panels F to J). Seeds containing embryos at the globular (A and F), heart (B and G), early-torpedo (C and H), torpedo (D and I) and maturation green (E and J) developmental stages were analyzed for GUS expression as described under Material and Methods. GUS expression in the root meristematic region of the embryo at heart (G) and early-torpedo (H) stages in *FPS2::GUS* seeds is indicated by arrowheads. The inset between panels (H) and (I) shows expression of *FPS2::GUS* in the root meristematic region and the pro-vascular tissue of an embryo at the early-torpedo stage.

Characterization of Arabidopsis *fps* single knockout mutants revealed a differential contribution of FPS isoforms to total FPS activity in mature seeds [26]. In order to investigate whether this effect could be due to differential expression of *FPS1* and *FPS2* in seed tissues, we analyzed the spatial and temporal pattern of expression of GUS activity in seeds at different developmental stages harvested from Arabidopsis lines harbouring *FPS1S::GUS* and *FPS2::GUS* transgenes (Figure 6). These chimeric gene fusions included approximately 1350 bp of the corresponding *FPS* promoter region [25]. In *FPS1S::GUS* plants, the primary site of GUS activity detection at all stages of development was the maternal chalazal seed coat (Figure 6A–E). At the globular, heart and early-torpedo stages of embryo development some variable and weaker GUS staining could also be detected in surrounding tissues (Figure 6A–C) although it is difficult to establish whether this expression reflected diffusion of the substrate or true expression of the transgene. At latter stages of embryo development GUS activity was restricted to the chalazal seed coat (Figure 6D and E). Interestingly, no expression of GUS activity in the embryo could be detected at any developmental stage. The pattern of GUS expression driven by the *FPS2* promoter was completely different (Figure 6F–J). At the globular stage of embryo development GUS staining was detected only in the chalazal endosperm (Figure 6F), but from the heart stage onward GUS activity could also be detected in the embryo (Figure 6G–J). At the heart and early torpedo stage GUS expression in the embryo was primarily localized to the root apical meristem region (Figure 6G and H, arrowhead) and the pro-vascular tissue of embryo (Figure 6, inset). Up to this developmental stage, a very faint staining could also be detected in the endosperm of some seeds when they were subjected to intensive staining. At later developmental stages GUS activity was clearly detected in the whole embryo as well as in the surrounding endosperm (Figure 6I). In mature seeds, strong GUS activity was also present in the cotyledonary embryo (Figure 6J), which is in sharp contrast to the absence of GUS activity in the embryo of mature seeds expressing *FPS1S::GUS* (Figure 6E). In conclusion, analysis of *FPS1S::GUS* and *FPS2::GUS* expression analysis demonstrated that *FPS* genes are differentially regulated during Arabidopsis seed development, showing highly complementary expression patterns particularly at late stages of seed formation.

### Functional Complementation of *fps2* Mutant Seed Phenotypes by FPS1S

**Figure 7:**
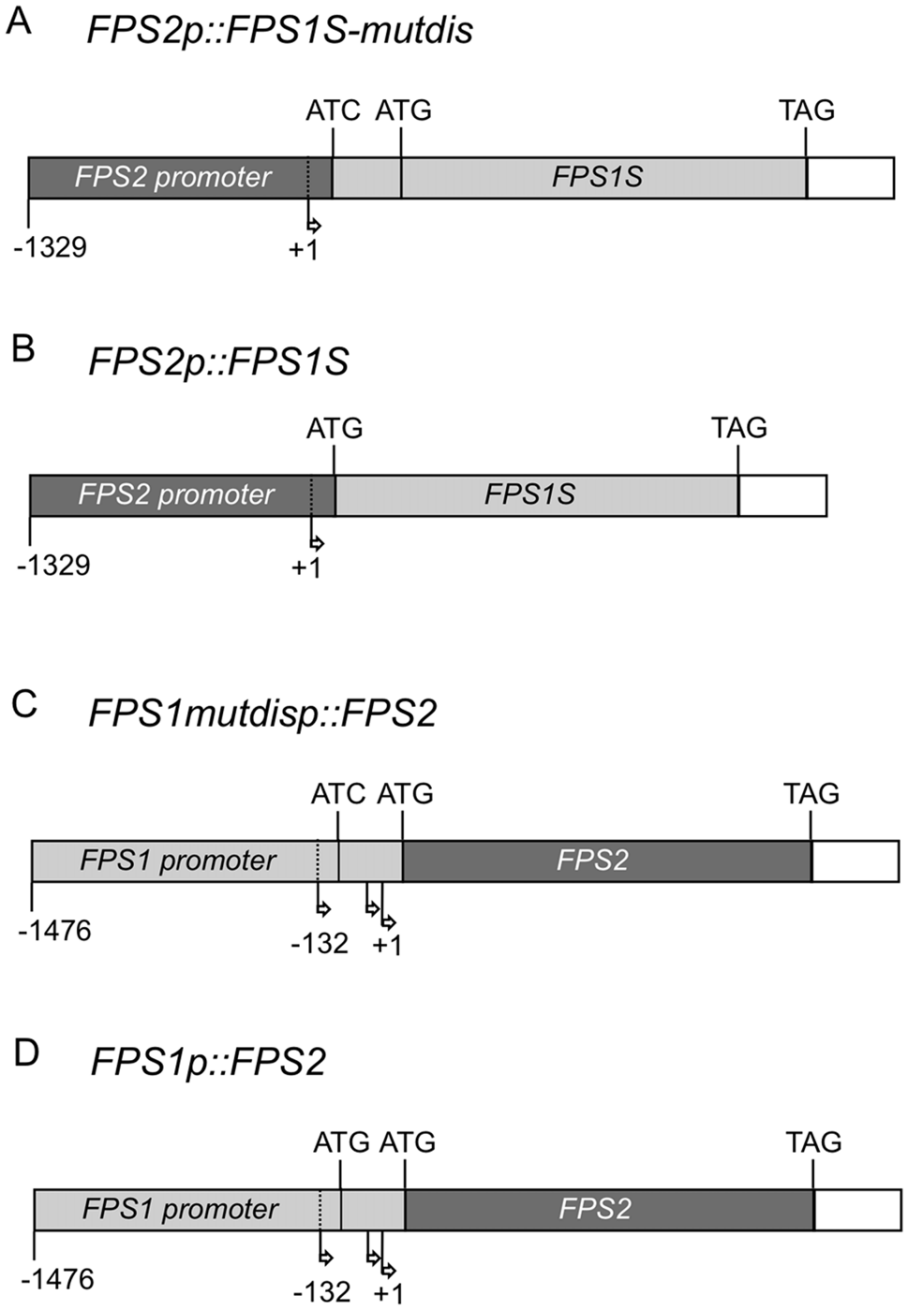
Schematic representation of chimeric genes *FPS2p::FPS1-mutdis* (A), *FPS2p::FPS1S* (B), *FPS1mutdisp::FPS2* (C) and *FPS1p::FPS2* (D). Dark gray boxes represent *FPS2* genomic sequences whereas sequences corresponding to *FPS1* gene are represented by light gray boxes. In all constructs the 3′-untranslated regions of both *FPS* genes is represented by an empty box. The position of the translational start codons is indicated as ATG and that of stop codons is indicated as TAG. In the *FPS2p::FPS1S-mutdis* (A) and *FPS1mutdisp::FPS2* (C) constructs, the 5′-most ATG codon in exon 1 of the *FPS1* gene was mutated to an ATC codon (encoding Ile). Arrows indicate the transcription start sites. In the *FPS1* gene, position +1 was assigned to the most internal transcription start site.

Mature seeds lacking FPS2 activity display several phenotypes including reduced levels of sitosterol, the main sterol found in plant tissues, increased HMGR activity and hypersensitivity to mevastatin compared to both *fps1* and wild-type seeds [26]. To determine whether FPS1S could complement these phenotypes, transgenic *fps2-1* plants expressing FPS1S under the control of the *FPS2* gene promoter were obtained. To this end we created two different gene constructs that were able to produce only isoform FPS1S (Figure 7A and B). The *FPS2p::FPS1S-mutdis* gene consisted of 1375 bp of the *FPS2* 5′-flanking region, including 1329 bp of the promoter and the entire 5′-untranslated region, fused to the genomic coding region of *FPS1* in which the distal ATG start codon was converted into an ATC codon to ensure that only FPS1S isoform could be produced. The *FPS2p::FPS1S* gene differed from *FPS2p::FPS1S-mutdis* gene in that the region comprised between the two ATG codons was removed.

**Figure 8:**
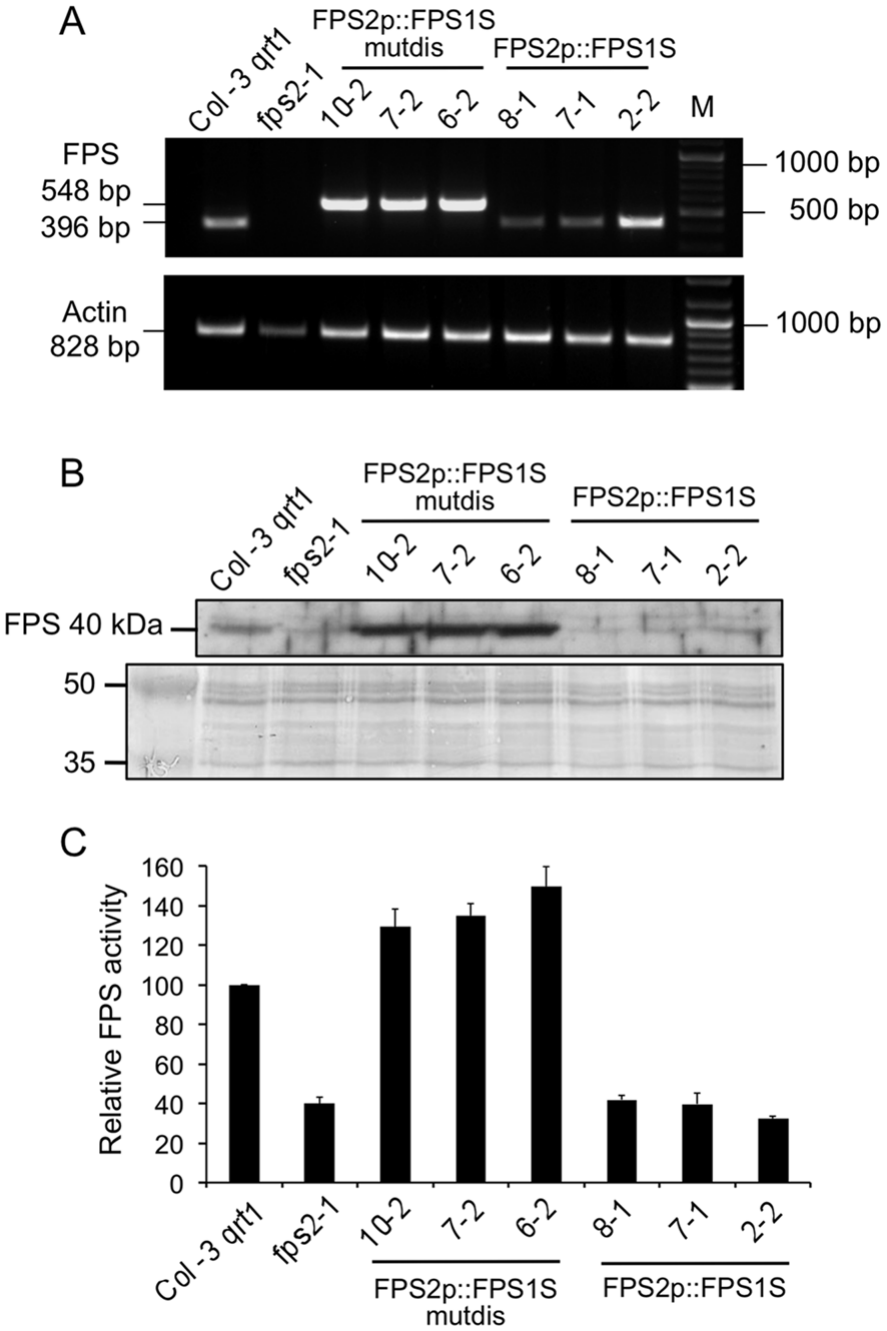
Characterization of *fps2-1* mutant lines harbouring *FPS2p::FPS1S* and *FPS2p::FPS1-mutdis* genes. (A) The expression of *FPS2p::FPS1S* and *FPS2p::FPS1-mutdis* was investigated using total RNA from 12-day-old seedlings of Arabidopsis wild-type, *fps2-1* and the indicated lines of the *fps2-1* mutant harbouring *FPS2p::FPS1S-mutdis* and *FPS2p::FPS1S* chimeric genes (upper panel). PCR products were electrophoresed in a 1% agarose gel. The size in bp of the amplified cDNA fragments corresponding to *FPS2p::FPS1-mutdis*, *FPS2p::FPS1S* and *ACT2* (actin) genes is indicated on the left. The size of the fragment amplified from *FPS2p::FPS1S-mutdis* lines (548 bp) was larger than that amplified from both *FPS2p::FPS1S* lines and wild-type plants (396 bp) because the FPS2::FPS1-mutdis mRNA contains the region between the two ATG translation start codons of the *FPS1* gene, which is not present in the FPS2::FPS1S mRNA. Numbers on the right indicate the sizes in bp of DNA markers shown in lane M. (B) Western blot analysis of total FPS protein in 16,000 *g* extracts from seeds of plant lines indicated above (upper panel). The lower panel shows the Coomassie blue-stained electrophoretic protein patterns in the 35 to 50 kDa range of extracts used for FPS protein level determinations. Images show the results of one representative experiment. (C) FPS activity in the 16,000 *g* protein extracts used for Western blot analysis. FPS activity in *fps* mutants is expressed relative to that in the wild-type, which was assigned a value of 100. The mean values and SE were calculated from three independent experiments.

**Figure 9:**
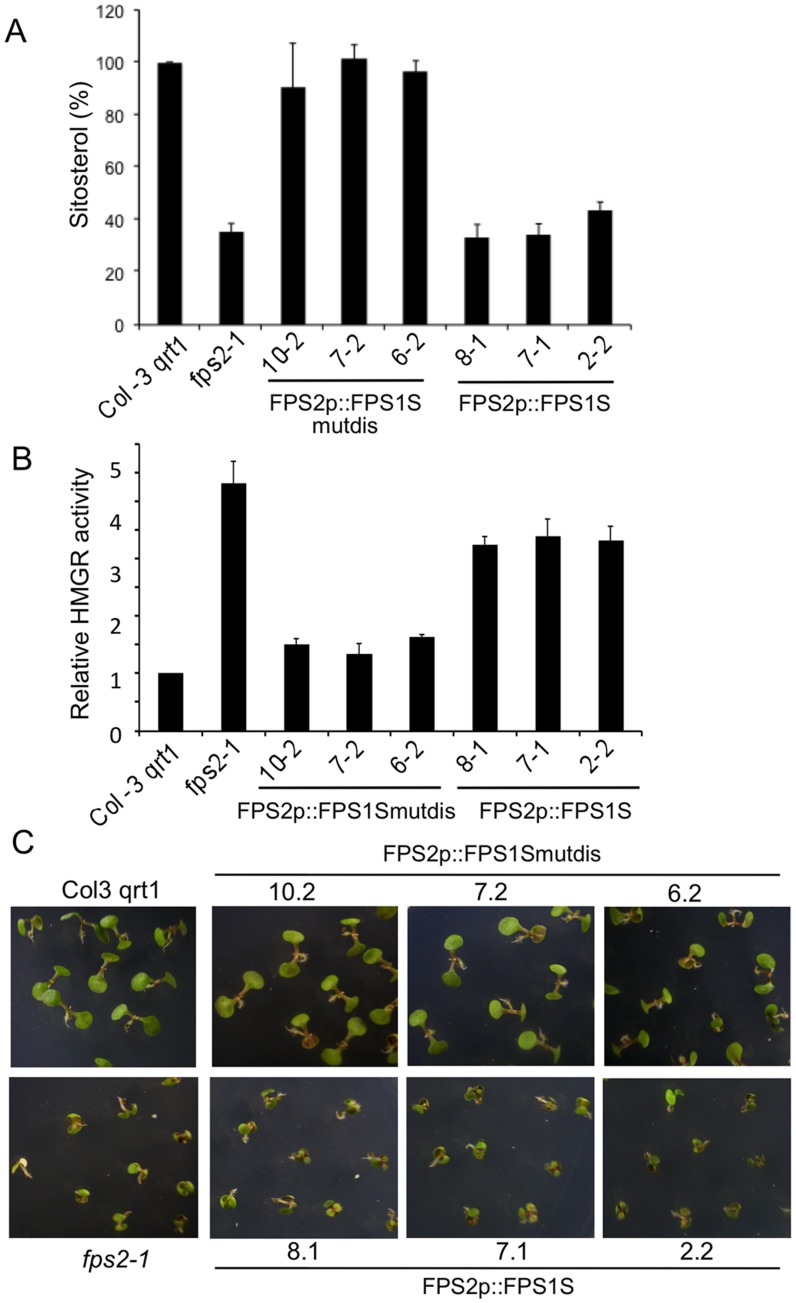
Reversion of *fps2-1* phenotypes by expressing FPS1S under control of the *FPS2* promoter. Sitosterol (A) and HMGR activity (B) levels in seeds from Arabidopsis wild-type, *fps2-1* and the indicated lines of the *fps2-1* mutant harbouring *FPS2p::FPS1S-mutdis* and *FPS2p::FPS1S* chimeric genes. Values are expressed relative to the wild-type values. Data represent the mean values and SE of three independent experiments. (C) Reversion of mevastatin hypersensitivity. Representative seedlings of the same lines were grown for 12 days under long-day conditions (16 h light/8 h dark) on MS plates supplemented with 1 µM mevastatin.

Several independent lines of these two kinds of transgenic plants were obtained and characterized. Among them, lines 6.2, 7.2 and 10.2 for *FPS2p::FPS1S-mutdis* and lines 2.2, 7.1 and 8.1 for *FPS2p::FPS1S* were selected for further characterization. The expression of *FPS2p::FPS1S-mutdis* and *FPS2p::FPS1S* was analyzed in young seedlings using semiquantitative RT-PCR and a primer set consisting of a forward primer specific for the *FPS2* 5′-untranslated sequence and a reverse primer located in the *FPS1* coding region. The three selected *FPS2p::FPS1S-mutdis* lines showed very similar levels of expression of the FPS1 mRNA that were even higher than those detected in wild-type plants (Figure 8A). Accordingly, protein extracts from seeds of *FPS2p::FPS1S-mutdis* lines also contained both higher levels of FPS1S protein, as demonstrated by Western blot analysis (Figure 8B), and higher values of FPS activity (Figure 9C) compared to wild-type seeds. On the contrary, FPS protein and enzyme activity levels in extracts from seeds of the three *FPS2p::FPS1S* transgenic lines were comparable to those found in extracts of *fps2-1* mutant seeds, which indeed were much lower that those detected in wild-type seed extracts (Figure 8B and C). The inability of *FPS2p::FPS1S* to restore wild-type levels of FPS protein and enzyme activity was not due to a lack of expression of the transgene, since its mRNA was detected in the seedlings of the three selected lines. In two of them (lines 7.1 and 8.1), expression of the transgene was less intense than in wild-type plants whereas in the third one (line 2.2) expression was slightly stronger than in control plants (Figure 8A). These observations suggested that translation of the chimeric FPS2::FPS1S mRNA expressed by *FPS2p::FPS1S* was severely impaired, which is most likely due to the different structural environment around the AUG start codon of the FPS2::FPS1S mRNA compared to that of the FPS2 and FPS2::FPS1S-mutdis mRNAs (Figure S1). Among the different structural features known to be involved in translational control of specific mRNAs [53], base-pairing involving nucleotides within the AUG codon has been recognized as an important structural determinant that may influence translation initiation [54], [55].

To complete the characterization of the transgenic lines we next assessed whether expression of FPS1S driven by the *FPS2* promoter could complement the characteristic phenotypes of *fps2-1* seeds. Sitosterol and HMGR activity levels were quantified in seeds of *FPS2p::FPS1S-mutdis* and *FPS2p::FPS1S* lines. None of the *fps2-1* seed phenotypes could be rescued by *FPS2p::FPS1S* expression (Figures 9A–C), a result that was fully consistent with the lack of FPS1S protein production and enzyme activity recovery (Figure 8B and C). On the contrary, sitosterol and HMGR activity levels were both restored to those of wild-type seeds in lines expressing *FPS2p::FPS1S-mutdis* (Figures 9A and B). Recovery of wild-type levels of HMGR activity in seeds also restored normal mevastatin sensitivity to these lines (Figure 9C). Altogether, these results demonstrated that FPS1S is able to functionally replace FPS2.

### Rescue of *fps2* Mutant Seed Phenotypes by Increasing Total FPS Activity in Seeds

**Figure 10:**
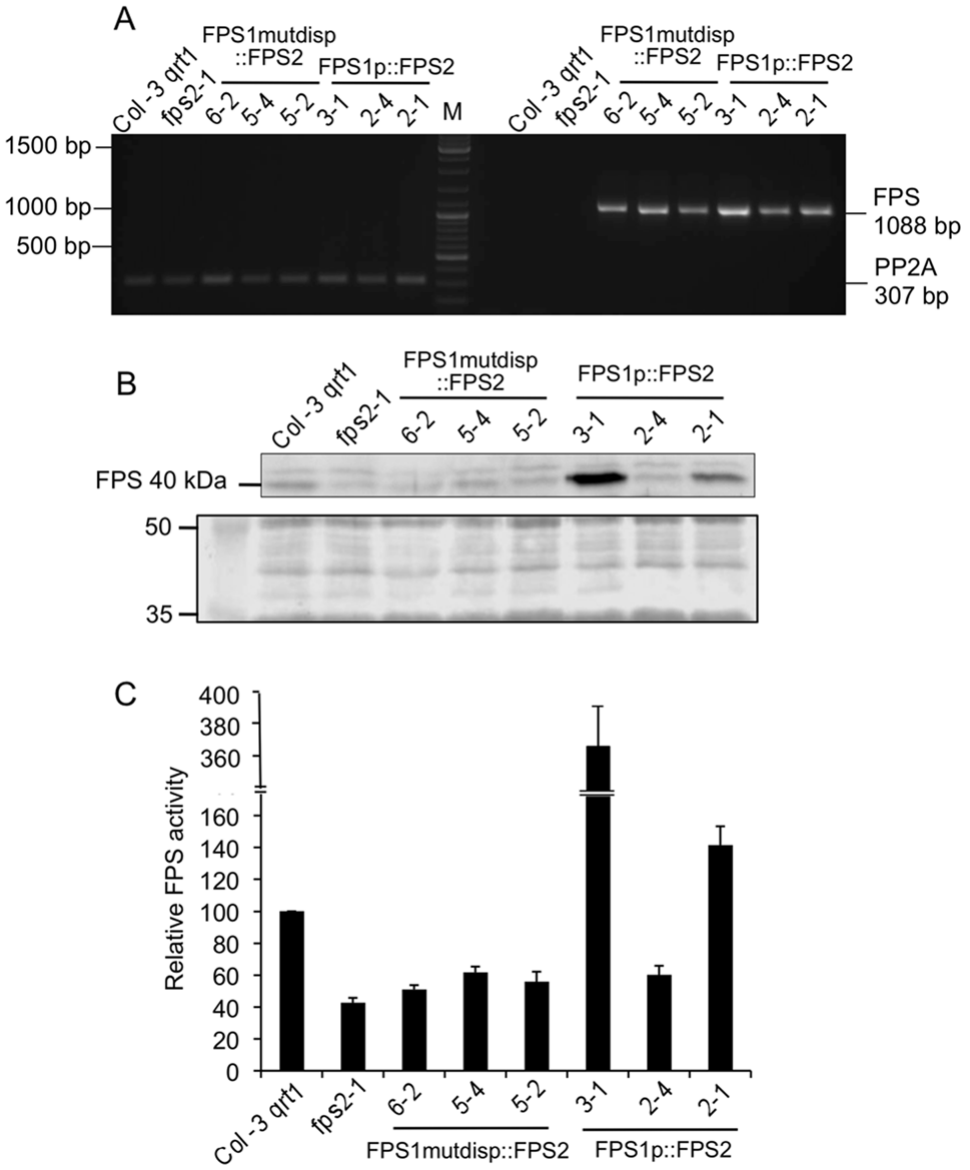
Characterization of *fps2-1* mutant lines harbouring *FPS1mutdisp::FPS2* and *FPS1p::FPS2* genes. (A) The expression of *FPS1mutdisp::FPS2* and *FPS1p::FPS2* was investigated using total RNA from 12-day-old seedlings of Arabidopsis wild-type, *fps2-1* and the indicated lines of the *fps2-1* mutant harbouring *FPS1mutdisp::FPS2* and *FPS1p::FPS2* chimeric genes. PCR products were electrophoresed in a 1% agarose gel. The size in bp of the amplified cDNA fragments corresponding to *FPS1mutdisp::FPS2* and *FPS1p::FPS2* (1088 bp) and *PP2A* genes (307 bp) is indicated on the right. Numbers on the left indicate the sizes in bp of DNA markers shown in lane M. (B) Western blot analysis of total FPS protein in 16,000 *g* extracts from seeds of plant lines indicated above (upper panel). The lower panel shows the Coomassie blue-stained electrophoretic protein patterns in the 35 to 50 kDa range of extracts used for FPS protein level determinations. Images show the results of one representative experiment. (C) FPS activity in the 16,000 *g* protein extracts used for Western blot analysis. FPS activity in mutants is expressed relative to that in the wild-type, which was assigned a value of 100. The mean values and SE were calculated from three independent experiments.

**Figure 11:**
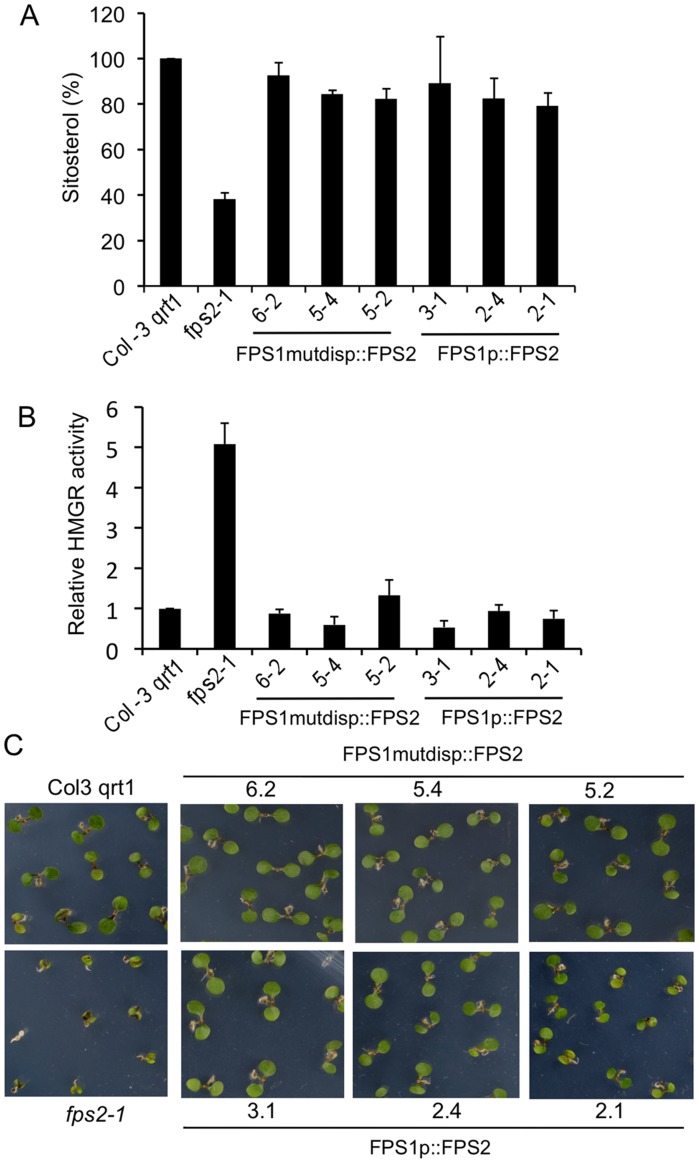
Reversion of *fps2-1* phenotypes by expressing FPS2 under the control of the *FPS1* promoter. Sitosterol (A) and HMGR activity (B) levels in seeds from Arabidopsis wild-type, *fps2-1* and the indicated lines of the *fps2-1* mutant harbouring *FPS1mutdisp::FPS2* and *FPS1p::FPS2* chimeric genes. Values are expressed relative to the wild-type values. Data represent the mean values and SE of three independent experiments. (C) Reversion of mevastatin hypersensitivity. Representative seedlings of the same lines were grown for 12 days under long-day conditions (16 h light/8 h dark) on MS plates supplemented with 1 µM mevastatin.

Once we had demonstrated that expression of FPS1S driven by the *FPS2* promoter was able to rescue the phenotypes caused by loss of function of FPS2, we set out to determine whether expression of FPS2 driven by the *FPS1* promoter could also rescue the *fps2-1* phenotypes. To this end *fps2-1* plants harbouring genes *FPS1mutdisp::FPS2* and *FPS1p::FPS2* (Figure 7C and D) were created and characterized. *FPS1mutdisp::FPS2* consisted of 1526 bp of the *FPS1* 5′-flanking region, including 1338 bp of the *FPS1* gene promoter and the entire 5′-untranslated region in which the distal ATG start codon was converted into an ATC codon, fused to the genomic coding region of *FPS2* so that only FPS2 isoform could be produced from this transgene. In the *FPS1p::FPS2* gene, the distal ATG codon was not disrupted and therefore this transgene could potentially express both FPS2 and a long version of FPS2 equivalent to FPS1L. Among the transgenic lines obtained, lines 6.2, 5.4 and 5.2 harbouring *FPS1mutdisp::FPS2*, and lines 3.1, 2.4 and 2.1 harbouring *FPS1p::FPS2* were selected for further characterization. The expression of the transgenes was analyzed in young seedlings of these lines using semiquantitative RT-PCR and a primer set consisting of a forward primer specific for the *FPS1* 5′-untranslated sequence and a reverse primer specific for the *FPS2* coding region. All selected lines showed comparable expression levels of the chimeric FPS2 mRNAs that, as expected, were not detected either in wild-type plants or in *fps2-1* plants (Figure 10A). However, striking differences in FPS protein and activity levels were observed between the two groups of transgenic plants. FPS protein content in seeds of the three *FPS1mutdisp::FPS2* lines was comparable and only slightly above that found in *fps2-1* seed extracts. By contrast, total FPS protein levels in *FPS1p::FPS2* seeds varied greatly. The amount of FPS protein in line 2.4 was pretty similar to that found in *FPS1mutdisp::FPS2* plants whereas line 3.1 had strikingly elevated levels of FPS protein. Line 2.1 showed intermediate levels of FPS that were, nevertheless, higher than in wild-type plants (Figure 10B). These differences could be due to the production of different relative amounts of short and long versions of FPS2 in *FPS1p::FPS2* seeds. Unfortunately, it is not possible to determine the relative contributions of the individual short and long FPS2 forms to the total amount of FPS2 protein since they both have the same size after processing of the long version of FPS2 [9]. In any case, a close correlation between FPS protein content and enzyme activity levels was observed in the seed extracts of all lines (Figure 10C). Analysis of the characteristic *fps2-1* phenotypes in seeds of the transgenic lines revealed that the phenotypes associated to loss of function of FPS2 were fully rescued in all transgenic lines regardless of the degree of FPS activity enhancement in the mature mutant seeds (Figure 11A–C). In fact, a small increase of FPS activity of only 1.2-fold (line 6.2) was sufficient to restore wild-type sitosterol and HMGR activity values as well as normal Mst sensitivity to the same extent than a drastic enhancement of FPS activity (approximately 9-fold in line 3.1). These results suggested that impaired synthesis of FPP in the mature embryo due to the lack of FPS2 activity could be fully compensated just by increasing slightly the synthesis of FPP in the seed maternal tissues.

## Discussion

Isozymes usually display specific biochemical properties that allow fine-tuning of metabolic pathways to meet the specific needs of tissues and organs at different developmental stages and/or under different environmental conditions. The occurrence in plants of small FPS isozyme families has raised the still-unanswered question about the role of individual FPS isozymes in the cytosol/ER isoprenoid biosynthetic pathway. Most of the information currently available on the biological function of FPS isozyme family members has been obtained from the pattern of expression of the corresponding genes [11], [14], [15], [16], [17], [25] and the characterization of mutants that either overexpress [8], [10] or lack individual FPS isozymes [26]. By contrast, much less attention has been paid to investigate and compare the biochemical properties of individual FPS isozyme family members, despite this knowledge may also greatly contribute to the understanding of their role in the isoprenoid pathway [18].

In this study we report a detailed functional and structural characterization of the Arabidopsis FPS1S and FPS2 isozymes. Both enzymes were efficiently expressed in *E. coli* as GST-FPS fusion proteins, though the GST tag was removed by proteolytic cleavage during the purification process (Figure 1) to avoid any interference it could have on FPS activity [56]. Biochemical characterization of the purified native FPS1S and FPS2 revealed that both enzymes displayed very similar pH-rate profiles with an optimum at pH 7.0 (Figure 2). This pH preference was consistent with the predicted cytosolic localization of both FPS isozymes because the pH of the cytoplasm of plant cells is kept slightly alkaline at 7.2-7.5 under non-eliciting conditions [57]. The cytosolic localization of both FPS isozymes was demonstrated by transiently expressing N- and C-terminal fusions of FPS1S and FPS2 with GFP in agroinfiltrated *N. benthamiana* leaf cells. Under our experimental conditions all the four fusion proteins localized exclusively in the cytosol (Figure 5). This result was fully consistent with the absence of canonical subcellular targeting signals in the FPS1S and FPS2 proteins, and argued against the possibility that FPS1S and FPS2 could harbour a cryptic signal for peroxisomal targeting as recently reported to occur in a *C. roseus* short FPS enzyme. In contrast to our results, an N-terminal fusion of CrFPS with YFP was found to localize both in the peroxisomes and the cytosol of *C. roseus* cells despite the protein does not contain any classical peroxisomal targeting sequence [21]. It is also worth noting that our finding that none of the Arabidopsis short FPS isoforms reside in the peroxisomes was fully consistent with the results of extensive proteomic studies that have detected both FPS1S and FPS2 in the cytosolic proteome of Arabidopsis [58] and could not detect any FPS protein in purified Arabidopsis peroxisomes [59], [60]. The distinct subcellular localization of Arabidopsis and *C. roseus* short FPS isozymes could be related to the different profile of isoprenoid compounds produced by these two plant species [3], [61], though the central position of FPS in the isoprenoid pathway and the fundamental role of isoprenoid biosynthesis in plant biochemistry and physiology would argue against this hypothesis. Thus, given the ongoing controversy regarding the subcellular localization of FPS and other MVA pathway enzymes and the potential interference of fluorescent protein tags with proper subcellular targeting of proteins, further studies based on alternative experimental approaches are required in order to establish whether or not short FPS isozymes localize in different subcellular compartments in a plant-species dependent manner.

Comparison of the steady-state kinetic constants of purified FPS1S and FPS2 (Table 1) revealed that both enzymes have similar *K*_m_ values for their substrates and a clear preference for GPP over DMAPP as the allylic substrate for FPP formation. A similar kinetic behaviour has also been reported for FPS enzymes from both eukaryotic and prokaryotic organisms [33], [18], [56], [62], [63]. The kinetic similarities between FPS1S and FPS2 are consistent with the high degree of sequence conservation (90.6% identity) shared by these isozymes. However, FPS1S and FPS2 also displayed some remarkable differential properties that must be a consequence of the small differences in their primary structure. FPS2 is a more efficient catalyst that FPS1S by a factor of 2.5- to 3-fold (Table 1). All known FPS proteins contain seven highly conserved amino acid motifs [64] that are involved in enzyme catalysis and product chain length determination [5], [65]. Arabidopsis FPS1S and FPS2 differ by only 32 amino acid residues out of a total of 343 and 342 amino acids, respectively. These amino acid changes are scattered throughout the sequence but, interestingly, none of them is located within the conserved motifs involved in catalysis [12]. Thus, it is difficult to attribute the differences in catalytic efficiency between FPS1S and FPS2 to a particular amino acid substitution. Rather it appears that this functional difference is the consequence of subtle changes in the three-dimensional structure of the enzymes due to multiple individual amino acid changes acting together. In fact, FPS is a homodimeric enzyme in which subunits interact with each other to form a shared active site rather than bearing an independent active site in each subunit [66]. The suggested structural differences between FPS1S and FPS2 must also account for the greater thermal stability (Figure 3) and higher sensitivity to the inhibitory effect of NaCl (Figure 2) exhibited by FPS2 compared to FPS1S. In fact, a single amino acid substitution is sufficient to alter temperature and salt sensitivity in the case of malate dehydrogenase [67], [68], [69]. Indeed, inspection of atomic-level homology models of FPS1S and FPS2 and theoretical calculations of the free energy and structural changes that accompany the single-site substitution of FPS2 unique amino acids into the FPS1S structural template indicate that surface salt bridge formation and reduced conformational entropy might largely account for the increased thermostability observed for FPS2. These stabilizing electrostatic effects are reflected in specific amino acid composition biases that have been extensively studied in thermophilic proteins [70], [71].

Our recent characterization of Arabidopsis *fps* single knockout mutants showing that FPS1S and FPS2 can almost fully compensate each other’s loss throughout most of the plant life cycle demonstrated that FPS1S and FPS2 have largely overlapping physiological functionalities. The only signs of functional specialization were observed in mature seeds and early developing seedlings. At these developmental stages FPS2 becomes the major contributor to total FPS activity, to the point that residual FPS1-derived activity in mature seeds is unable to supply enough FPP for normal sterol production, which triggers a compensatory enhancement of HMGR activity that is crucial for proper seed germination and seedling establishment [26]. Interestingly, our GUS expression analysis during seed development revealed a marked spatial and temporal divergence in the *FPS1* and *FPS2* expression pattern, which from the torpedo stage onward showed a strong degree of qualitative complementarity (Figure 6), also known as reciprocal expression pattern. This is when only one gene copy is expressed in some organs or tissue types and the other copy is expressed in others [72]. In the mature stages of seed development *FPS2* expression clearly dominates over the expression of *FPS1*. The *FPS2* gene is expressed throughout the endosperm and the embryo at the torpedo stage, and throughout the cotyledonary embryo in mature seeds, whereas the expression of *FPS1* remains restricted to the maternal chalazal seed coat. The exclusive expression of *FPS2* in the whole cotyledonary embryo, which at this stage occupies most of the seed, along with the higher catalytic efficiency of FPS2, explain why this isozyme is the major contributor to total FPS activity in mature seeds [26]. Interestingly, our results from genetic cross-complementation studies of the *fps2-1* mutant seed phenotypes showed that expression of FPS1S driven by the *FPS2* gene promoter (Figure 8) restored wild-type sitosterol and HMGR activity levels as well as normal sensitivity to mevastatin (Figure 9). This finding demonstrated that under normal conditions FPS1S and FPS2 are completely functionally interchangeable, which is fully consistent with the kinetic similarities exhibited by FPS1S and FPS2, and raises the question as to why FPS2 is the predominant FPS isozyme expressed in mature seeds and during the early stages of seed germination and seedling emergence. Seed viability is essential for survival of higher plants and therefore seeds are well equipped to withstand extended periods of exposure to harsh environmental conditions, as for instance extreme temperatures that may cause protein denaturation and inactivation among other effects. Arabidopsis seeds can complete germination even after treatment for more than 3.5 hours at 45°C [73], a temperature that is lethal for seedlings [74]. It is thus tempting to speculate that FPS2 has been evolutionarily selected as the predominant FPS isozyme in mature seeds of Arabidopsis because of its striking thermotolerance that could contribute to maintain seed germination capability of seeds exposed to high temperatures. It will be interesting to determine whether other isoprenoid biosynthetic enzymes expressed in seeds also display this biochemical feature. Moreover, the higher catalytic efficiency of FPS2 compared to FPS1S would enable early developing seedlings to sustain an active synthesis of isoprenoid precursors until the newly made FPS1S replaces FPS2 in this task.

FPP synthesis has been found to be indispensable for Arabidopsis embryos to progress beyond the pre-globular/globular stage and continue further development [26]. Our GUS expression analysis in seeds suggests that early developing embryos do not synthesize their own FPP since no embryo-specific expression of any of the *FPS* genes could be detected until the heart stage of embryo development (Figure 6). Rather it seems that early developing embryos import FPP or downstream FPP-derived isoprenoid precursors from other seed tissues like the maternal chalazal seed coat and/or the chalazal endosperm, where FPP can be synthesized as inferred from the expression of *FPS1* and *FPS2* in these tissues. This is a plausible hypothesis since the maternal chalazal seed coat and the chalazal endosperm are both considered to be specialized seed tissues involved in the uptake, reprocessing and release of metabolites into the endosperm to support embryogenesis [75], [76]. The proposal that an active synthesis of isoprenoid precursors occurs in the chalazal endosperm at the early stages of embryo development is further supported by the results of a previous study showing a strong expression of isopentenyl transferase (IPT) genes *AtIPT4* and *AtIPT8* in the chalazal zone of Arabidopsis seeds, that disappeared when the embryo progressed to the heart stage [77]. IPTs catalyze the first committed step of the cytokinin biosynthetic pathway, the isopentenylation of AMP, ADP and ATP from DMAPP [78]. Interestingly, the finding that *FPS2* gene started to be expressed in embryos at the heart stage of development suggests that the embryo acquires the ability to synthesize its own FPP at this stage, though this biosynthetic capability does not seem to be essential for normal embryo and seed development since embryos in *fps2* mutant seeds lacking FPS2 activity are fully viable (Figure S2). These observations support the hypothesis that the maternal chalazal seed coat is able to supply sufficient FPP or FPP-derived precursors to the embryo and the endosperm to sustain normal seed development, though the amount of isoprenoid precursors supplied by this tissue would not be sufficient to sustain normal sterol production in the *fps2* seeds (Figure 9). The finding that expression of FPS2 under control of the *FPS1* promoter (Figure 10), whose activity is restricted to the chalazal seed coat based on the GUS expression analysis (Figure 6), was also able to restore normal sterols levels to *fps2* seeds (Figure 11) lends further support to the hypothesis that the maternal chalazal seed coat can function as a source of FPP for the developing embryo and reinforces the view that maternal seed tissues contribute sterol precursors to developing Arabidopsis embryos [79]. Nevertheless, to the best of our knowledge direct experimental evidence of FPP exchange between cells has yet to be provided. These cross-complementation experiments also indicated that the level of FPS1-derived activity in the chalazal seed coat of *fps2* seeds is only slightly below the minimum threshold of total FPS activity needed to sustain a normal flux through the sterol pathway in seeds, since a 1.2-fold increase of FPS activity in this tissue with respect to the activity in *fps2* seeds (Figure 10) is enough to prevent all *fps2* seed and seedling phenotypes including normal sterols levels (Figure 11). In conclusion, the existence of two potential sources of FPP in seeds would explain why under normal conditions embryos and seeds of *fps* single knockout mutants develop like their wild-type counterparts.

## Supporting Information

Figure S1 **Predicted secondary structure of the region containing the AUG translation initiation codon of the FPS2::FPS1S, FPS2, and FPS2::FPS1S-mutdis mRNAs.** Secondary structure models were generated by using the RNAfold web server (http://rna.tbi.univie.ac.at/). The AUG start codons are marked with an oval.
(TIF)

Figure S2 **Normal embryo development in** ***fps2-1*** **mutant seeds.** Seeds with embryos at the indicated developmental stages were fixed and cleared for visualization as previously described [26].
(TIF)
