# Supplementary figures and images for: Characterization of Arabidopsis FPS Isozymes and FPS Gene Expression Analysis Provide Insight into the Biosynthesis of Isoprenoid Precursors in Seeds
Source: PLoS One. 2012 Nov 7;7(11):e49109. doi: 10.1371/journal.pone.0049109 (PMC3492304; doi:10.1371/journal.pone.0049109)

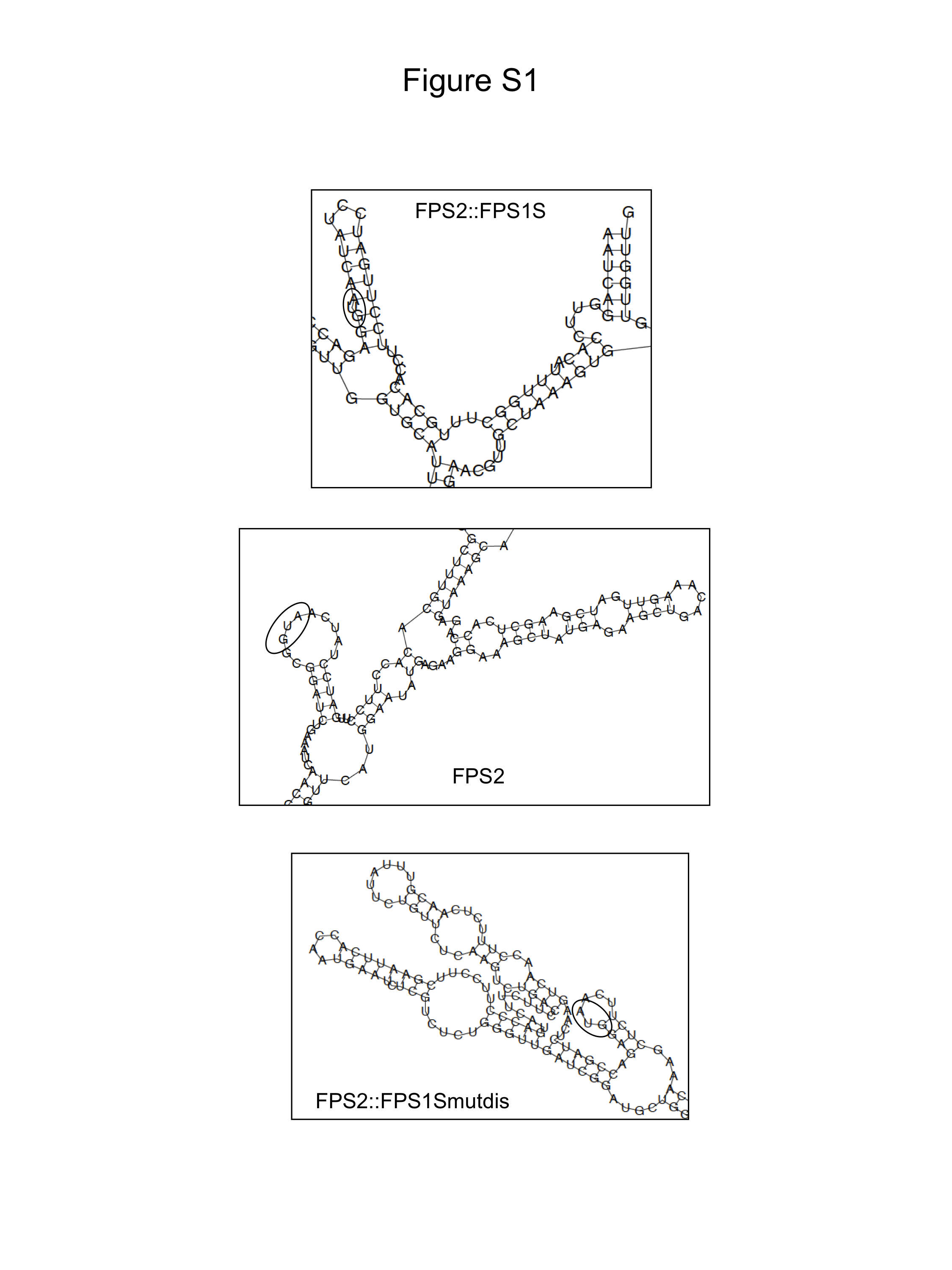

Supplement: Figure S1 — Predicted secondary structure of the region containing the AUG translation initiation codon of the FPS2::FPS1S, FPS2, and FPS2::FPS1S-mutdis mRNAs. Secondary structure models were generated by using the RNAfold web server (http://rna.tbi.univie.ac.at/). The AUG start codons are marked with an oval. (TIF) [file pone.0049109.s001.tif]
